# Changes in metabolite profiles in the cerebrospinal fluid and in human neuronal cells upon tick-borne encephalitis virus infection

**DOI:** 10.1186/s12974-025-03478-4

**Published:** 2025-06-14

**Authors:** Satoshi Suyama, Sally Boxall, Benjamin Grace, Andrea Fořtová, Martina Pychova, Lenka Krbkova, Rupasri Mandal, David Wishart, Diane E. Griffin, Daniel Růžek, Niluka Goonawardane

**Affiliations:** 1 School of Molecular and Cellular Biology, Faculty of Biological Sciences, and Astbury Centre for Structural Molecular Biology, University of Leeds, Leeds, LS2 9JT UK; 2 Queens’ College, University of Cambridge, Cambridge, CB3 9ET UK; 3 Institute of Experimental Biology, Faculty of Science, Masaryk University, Kamenice 735/5, Brno, CZ-62500 Czechia; 4 Institute of Parasitology, Biology Centre of the Czech Academy of Sciences, Ceske Budejovice, CZ-62100 Czechia; 5 Veterinary Research Institute, Brno, CZ-62100 Czechia; 6 Department of Biological Sciences and Computing Science, University of Alberta, Edmonton, AB Canada; 7 Department of Infectious Diseases, Faculty of Medicine, University Hospital Brno, Masaryk University, Brno, Czechia CZ-62500 Czechia; 8 W. Harry Feinstone Department of Molecular Microbiology and Immunology, Johns Hopkins Bloomberg School of Public Health, 615 N. Wolfe St, Rm E5636, Baltimore, MD 21205 USA; 9 Department of Children’s Infectious Disease, Faculty of Medicine and University Hospital, Masaryk University, Brno, Czechia CZ-61300 Czechia

**Keywords:** Tick-borne encephalitis virus, Cerebrospinal fluid, Human motor neurons, Metabolomics, Pro-inflammatory cytokines, Chemokines, Neuroinflammation

## Abstract

**Background:**

Tick-borne encephalitis virus (TBEV) is a significant threat to human health. The virus causes potentially fatal disease of the central nervous system (CNS), for which no treatments are available. TBEV infected individuals display a wide spectrum of neuronal disease, the determinants of which are undefined. Changes to host metabolism and virus-induced immunity have been postulated to contribute to the neuronal damage observed in infected individuals. In this study, we evaluated the cytokine, chemokine, and metabolic alterations in the cerebrospinal fluid (CSF) of symptomatic patients infected with TBEV presenting with meningitis or encephalitis. Our aim was to investigate the host immune and metabolic responses associated with specific TBEV infectious outcomes.

**Methods:**

CSF samples of patients with meningitis (*n* = 27) or encephalitis (*n* = 25) were obtained upon consent from individuals hospitalised with confirmed TBEV infection in Brno. CSF from uninfected control patients was also collected for comparison (*n* = 12). A multiplex bead-based system was used to measure the levels of pro-inflammatory cytokines and chemokines. Untargeted metabolomics followed by bioinformatics and integrative omics were used to profile the levels of metabolites in the CSF. Human motor neurons (hMNs) were differentiated from induced pluripotent stem cells (iPSCs) and infected with the highly pathogenic TBEV-Hypr strain to profile the role(s) of identified metabolites during the virus lifecycle. Virus infection was quantified via plaque assay.

**Results:**

Significant differences in proinflammatory cytokines (IFN-α2, TSLP, IL-1α, IL-1β, GM-CSF, IL-12p40, IL-15, and IL-18) and chemokines (IL-8, CCL20, and CXCL11) were detected between neurological-TBEV and control patients. A total of 32 CSF metabolites differed in TBE patients with meningitis and encephalitis. CSF S-Adenosylmethionine (SAM), Fructose 1,6-bisphosphate (FBP1) and Phosphoenolpyruvic acid (PEP) levels were 2.4-fold (range ≥ 2.3-≥3.2) higher in encephalitis patients compared to the meningitis group. CSF urocanic acid levels were significantly lower in patients with encephalitis compared to those with meningitis (*p* = 0.012209). Follow-up analyses showed fluctuations in the levels of O-phosphoethanolamine, succinic acid, and L-proline in the encephalitis group, and pyruvic acid in the meningitis group. TBEV-infection of hMNs increased the production of SAM, FBP1 and PEP in a time-dependent manner. Depletion of the metabolites with characterised pharmacological inhibitors led to a concentration-dependent attenuation of virus growth, validating the identified changes as key mediators of TBEV infection.

**Conclusions:**

Our findings reveal that the neurological disease outcome of TBEV infection is associated with specific and dynamic metabolic signatures in the cerebrospinal fluid. We describe a new in vitro model for in-depth studies of TBEV-induced neuropathogenesis, in which the depletion of identified metabolites limits virus infection. Collectively, this reveals new biomarkers that can differentiate and predict TBEV-associated neurological disease. Additionally, we have identified novel therapeutic targets with the potential to significantly improve patient outcomes and deepen our understanding of TBEV pathogenesis.

**Supplementary Information:**

The online version contains supplementary material available at 10.1186/s12974-025-03478-4.

## Background

Tick-borne encephalitis virus (TBEV) is an *Ixodes* spp.-tick-borne orthoflavivirus with the potential to cause human epidemics of severe and fatal encephalitis [1–3]. TBEV is endemic in Asia (including northern China and Japan) [4, 5] and in 27 countries in Europe [1, 6–8], including recent detection in the UK [9], Netherlands [10] and North Africa [11]. TBEV is recognised as a global pathogen of concern and is highlighted in the World Health Organisation (WHO) global vector control response 2017–2030. TBEV is classified into five subtypes: Baikalian, Himalayan, European, Siberian, and Far Eastern [12].

TBEV primarily infects neurons and is associated with CNS pathology [3, 13–16]. Human TBEV is acquired through the bite of an infected tick or through the consumption of unpasteurized milk or milk products from infected animals (alimentary transmission). TBEV associated disease follows a biphasic course; Phase 1 (viraemic) is characterised by non-specific febrile illness (flu-like symptoms), lasting up to 7 days; Phase 2 (∼ 30% of those infected) involves neurological disease (tick-borne encephalitis [TBE]) [17–20] that manifests as a range of nonspecific symptoms including meningitis, encephalitis, loss of coordination, difficulties with speech, limb weakness, seizures, fever and headaches [2, 17, 20–24]. Severe TBEV cases are associated with cytopenia, elevated serum C-reactive protein (CRP) levels in the blood, serum anti-TBEV IgG antibodies [2, 17] and low levels of neutralizing antibodies in both the serum and CSF [22]. Neurological manifestations are less well understood, with no specific CNS biomarkers identified.

The clinical course and outcome of TBEV infection is dictated by the site of infection, host immune response, genetics, virus subtype/strain and age [2, 20, 25]. The incubation period ranges from 7 to 14 days, but shorter periods (3–4 days) have been reported for alimentary infections [8, 26, 27]. Severe forms of higher susceptibility have been described in adults (> 60 years of age) with those aged over 40 years at an increased risk of developing encephalitis [2, 17, 21]. Approximately 40–50% of adults (age ranged 16–76) develop post-encephalitic syndrome [28] that can last up to 18 months after the onset of the acute (first phase) illness. Over 10% of those infected show permanent sequelae, including spinal nerve paresis, dysarthria and severe mental disorders [28–30]. Age-dependent (range 15–78) long*-*term or permanent neuropsychiatric disorders are observed in ∼ 20% of those infected [13].

Given the complexity of disease presentation, the discovery of biomarkers that can predict disease progression hold great value for TBEV studies. Metabolomic analysis of the cerebrospinal fluid (CSF) has increased knowledge of the naturally occurring processes of virus infections including for COVID-19 [31], West Nile [32] and dengue fever [33] and can reflect disease-associated CNS pathology [31, 34–36]. Metabolism is also closely linked to the immune response to virus infection, with succinate, glucose and specific lipids identified as proinflammatory (e.g., IL-1β) and itaconate an anti-inflammatory (negative regulator of IL-6 and IL-12) [31, 37].

In this study, to address knowledge gaps regarding the outcome of TBEV-associated disease, we characterised key changes in metabolites, proinflammatory cytokines and chemokine levels in the CSF samples of hospitalised TBEV patients. As the patient cohorts were documented for TBEV-associated neurological outcomes, this allowed us to identify key CSF metabolites related to disease severity that could be used to predict the course of TBEV infection. A new physiological in vitro model of TBEV-infection was also developed using human iPSC-derived motor neurons to confirm the role of identified metabolites during the TBEV-lifecycle. Herein, we report the identification of inflammatory and metabolic markers for the diagnosis of TBEV in CSF samples that can stratify patients with encephalitis and meningitis.

## Materials and methods

### Clinical information and sample collection

Cerebrospinal fluid (CSF) samples were obtained upon consent from individuals hospitalised with confirmed TBEV (strain Hypr) or with non-TBEV in Brno (approval no. 103/19), Czech Republic. Samples were from the recent seasonal TBEV outbreak (2020–2023). Protocols were approved by the ethics committee of the University Hospital in Brno (date of approval: June 27, 2018). Clinical data were obtained from hospitalised CNS-TBEV patients at the treating hospital. TBEV clinical tests were conducted using EIA TBE Virus IgG (TBG096) and EIA TBE Virus IgM (TBM096) kits from TestLine Clinical Diagnostics. Severity of disease was evaluated according to the following scale: [1] Mild, flu-like symptoms with meningeal irritation defined as meningitis, characterised by fever, fatigue, nausea, headache, back pain, arthralgia/myalgia and neck or back stiffness; [2] Moderate, previous symptoms together with tremor, vertigo, somnolence and photophobia defined as meningoencephalitis; [3] Severe, prolonged neurological consequences including ataxia, titubation, altered mental status, memory loss, quantitative disturbance of consciousness, and palsy revealed as encephalitis, encephalomyelitis, or encephalomyeloradiculitis. Samples were collected through lumbar puncture (LP) and stored at − 80 °C prior to analysis.

A total of 64 participants were enrolled, including 25 diagnosed with encephalitis, 27 with meningitis (Supplementary Tables S1 and S2), and 12 with non-TBEV associated illness (control group). The control group did not present any neurological disease and were not diagnosed with known virological infections. Samples were obtained at the time of hospitalisation during the second phase of the disease. Four-month follow-up samples were collected from 10 cohorts, comprising 6 individuals diagnosed with encephalitis and 4 with meningitis. Patient demographics, clinical outcomes, immune responses (cytokines/chemokines) and metabolomics of CSF samples were analysed to fully profile the host response to TBEV infection.

### RNA extraction

Total RNA was isolated using ZymoBIOMICS DNA/RNA Miniprep Kit (ZYMO RESEARCH, R2002), accordingly to the manufacturers protocol. RNA was eluted, quantified (NanoDrop Microvolume Spectrophotometers, Thermo Fisher Scientific) and stored at − 80 °C.

### RT-PCR

RT–PCRs were performed as previously described [7, 38]. Briefly, One-Step TB Green PrimeScript RT-PCR Kit II (Takara, RR086A) was used. A total of 1 µg of total RNA was purified from the CSF. Analysis was performed using primers at a concentration of 0.8 µM in a final volume of 25 µl. One step RT-PCR was performed at 42 °C for 5 min, followed by denaturation (95 °C/5 secs) and 40 cycles of amplification with denaturation at 95 °C for 5 s and annealing at 60 °C for 30 s. Ct value ≥ 40 was considered as a cut-off for no virus detection. Statistical analysis was performed using GraphPad Prism 9 software.

### Chemokine measurements in CSF samples from TBEV-patients and controls

The levels of 13 proinflammatory chemokines in the CSF (25 µL) were simultaneously measured using LEGENDplex™ HU Proinflam. Chemokine Panels (740984; BioLegend), according to the manufacturer’s instructions. Analysis was performed on a Cytoflex machine. The human proinflammatory chemokine panel included MCP-1 (CCL2), RANTES (CCL5), IP-10 (CXCL10), Eotaxin (CCL11), TARC (CCL17), MIP-1α (CCL3), MIP-1β (CCL4), MIG (CXCL9), MIP-3α (CCL20), ENA-78 (CXCL5), GROα (CXCL1), I-TAC (CXCL11) and IL-8 (CXCL8). Data were analysed using BioLegend’s LEGENDplex™ (LEGENDplex™ Software (biolegend.com) or FlowJo (BD) (FlowJo, LLC). Statistical analysis was performed using GraphPad Prism 9 software.

### Cytokine measurements in CSF samples from TBEV-patients and controls

The levels of 13 cytokines in the CSF were measured using the LEGENDplex™ Human Cytokine Panel (741377; BioLegend) on a Cytoflex machine. The human proinflammatory chemokine panel included IFN-α2, TSLP, IL-1α, IL-1β, GM-CSF, IL-11, IL-12p40, IL-12p70, IL-15, IL-18, IL-23, IL-27 and IL-33. Data were analysed using BioLegend’s LEGENDplex™ (LEGENDplex™ Software (biolegend.com) or FlowJo (BD) (FlowJo, LLC). Statistical analysis was performed using GraphPad Prism 9 software.

### Untargeted polar metabolomic profiling for CSF samples

A total of 50 µL of CSF was mixed in 80% methanol (Sigma) on dry ice and incubated at − 80 °C for 4 h. CSF were centrifuged at 14,000 rfc for 20 min at 4 °C. Supernatants were extracted and stored at − 80 °C. The Weill Cornell Medicine Meyer Cancer Center Proteomics & Metabolomics Core Facility performed hydrophilic interaction liquid chromatography-mass spectrometry (LC-MS) for the relative quantification of polar metabolite profiles. Metabolites were measured on a Q Exactive Orbitrap mass spectrometer, coupled to a Vanquish UPLC system using an Ion Max ion source with a HESI II probe (Thermo Scientific). A Sequant ZIC-pHILIC column (2.1 mm i.d. × 150 mm, particle size of 5 μm, Millipore Sigma) was used for separation. MS data were processed using XCalibur 4.1 (Thermo Scientific) to obtain metabolite signal intensities for relative quantitation. For untargeted metabolomics, metabolites were identified by mass by matching of the MS signal to metabolites in the HMDB database. If multiple metabolites were matched to a specific MS signal, all were grouped into a single identification and ordered based on the number of references included in the HMDB database (high to low). The first ranked metabolite was used for downstream analyses. When multiple values with the same metabolite were attributed (different metabolites with same mass and retention time), all were used to avoid bias for which intensities/attributions were considered. Peak intensities for metabolites were screened for missing values. Relative abundances were analysed using MetaboAnalyst software version 6.0 [39]. Significant differences in metabolites were determined using a one-way ANOVA with post-hoc t-test. Cut-off values were raw *p* value < 0.05. The pathway significance cut-off was FDR corrected *p* value < 0.05.

### Metabolite network analysis

Metabolites pairs with Pearson correlation coefficients ≥ 0.6 and correlation *p*-values < 0.05 were shown as significantly associated. Regression analysis and correlation calculations were performed in R. Pathway enrichment analysis was performed using the Kyoto Encyclopedia of Genes and Genomes (KEGG) was performed using the ClueGO plugin (Cytoscape App Store - ClueGO). This integrates KEGG pathways and other ontologies to create functionally grouped networks. Connected metabolites were visualized as networks in Cytoscape (https://cytoscape.org). For Enrichment analysis and visualisation, Enrichment Map v.3.0; clusterMaker2, v.0.9.5; WordCloud, v.3.1.0; and AutoAnnotate, v,1.2.0, were used with the Cytoscape desktop application. All can be downloaded and installed at the EnrichmentMap Pipeline Collection (http://apps.cytoscape.org/apps/enrichmentmappipelinecollection).

### Human induced pluripotent stem cell (hiPSC) culture

Healthy Control Human iPSC Line SCTi003-A (Catalog # 200–0511) were purchased from StemCell Technologies. Cells were expanded on hESC-Qualified Matrigel (Corning, 354277) in mTeSR1 medium (StemCell Technologies, 85850). hiPSC cells were maintained in mTeSR1, Ak04CT (Ajinomoto, StemFit Ak04CT) or Cellartis^®^ DEF-CS™ 500 COAT-1 (Takara, Y30012) media. iPSCs were seeded 1/50 on hESC-Qualified Matrigel coated 6 well plates every 7 days. Culture media was changed every other day. In Ak04CT medium, 13,000 cells iPSCs were seeded onto iMatrix 511 Silk (nippi) coated 6 well plates every 7 days. Culture media was changed every other day. In Cellartis^®^ DEF-CS™ 500 COAT-1 medium, iPSCs were seeded (5.0 × 10^4^ cells/cm^2^ density) using COAT-1 reagent. Culture media was changed every day. iPSCs were assessed by qPCR. Primers used are listed in Table 1.

**Table 1:** Primers used in the study

Nanog_F	CAGGTGTTTGAGGGTAGCTC
Nanog_R	CGGTTCATCATGGTACAGTC
Sox2_F	TAGAGCTAGACTCCGGGCGATGA
Sox2_R	TTGCCTTAAACAAGACCACGAAA
Oct_F	TCTTTCCACCAGGCCCCCGGCTC
Oct_R	TGCGGGCGGACATGGGGAGATCC
cMyc_F	TGACCTAACTCGAGGAGGAGCTGGAATC
cMyc_R	AAGTTTGAGGCAGTTAAAATTATGGCT GAAGC
klf4_F	AGTGTGACAGGGCCTTTTCCAGGT
klf4_R	AAGCTGACTTGCTGGGAACTTGACC
beta-Actin_F	CGTGGGCCGCCCTAGGCACCA
beta-Actin_R	TTGGCCTTAGGGTTCAGGGGG
GAPDH_F	GCACAGTCAAGGCCGAGAAT
GAPDH_R	GCCTTCTCCATGGTGGTGAA
HPRT_F	GCACAGTCAAGGCCGAGAAT
HPRT_R	GCCTTCTCCATGGTGGTGAA

### Motor neuron generation and expansion

Protocols were adapted from previously published methods [40, 41]. hiPSCs were maintained with Ak04CT and seeded at 2.5 × 10^4^ cells/cm^2^ density on iMatrix511 Silk. After 3-days of culture, media was changed to hMN neural progenitor cell differentiation media (Advanced DMEM/F-12, 2% B27 supplement minus vitamin A, 100 U/mL Penicillin-Streptomycin, 150 nM LDN193189, 5 µM SB431542, 3 µM CHIR99021 and 1µM Retinoic Acid (Sigma, R2625). hMN differentiation Media supplemented with 1 µM purmorphamine was changed at days 2 and 4 post-differentiation. At Day 5, polystyrene plates were coated with 0.1 mg/mL PDL. At Day 6, PDL coated dishes were coated with iMatrix-511 slik and Fibronectin for 1 h. Differentiated NPCs were collected with Accutase. At this stage, NPC stock cryovials were prepared with Bambanker. NPCs were seeded at a density of 5 × 10^4^ cells/cm^2^ in human motor neuronal differentiation media (Advanced DMEM/F-12, 2% B27 supplement minus vitamin A, 100 U/mL Penicillin-Streptomycin, 3 µM CHIR99021, 1 µM Retinoic Acid, 1 µM purmorphamine, 200 µM Ascorbic Acid, 200 µM dbcAMP and 20 µM DAPT). At day 6, 10 µM Y27632 was added. Differentiation media was changed at days 7, 9 and 11. From day 13, media was changed every other day with 3 µM CHIR99021, 1 µM Retinoic Acid and 1 µM purmorphamine removed from the differentiation media. From day 18, DAPT was also removed. hMNs were assessed by qPCR. Primers used are listed in Table 1.

### Immunofluorescence assays

The expression of Nanog (R&D Systems; AF1997), Oct-3/4 (R&D Systems; AF1759), beta-III Tubulin (TuJ-1) (Abcam; ab18207), HB9 (Stratech; ORB157435-BOR) and TBEV envelope (E) glycoprotein (Absolute antibody; Ab03172-1.1-BT [T025]) were evaluated by immunofluorescence (IF), as described previously [38]. Briefly, iPSCs or neurons were fixed in 4% paraformaldehyde for 20 min, washed with PBS, permeabilized using 0.5% Triton X in PBS and blocked in 5% BSA in PBS. Primary and fluorochrome-conjugated secondary antibodies (Thermo Fisher; 1:500 dilution) were added in 1% BSA in PBS. Confocal images were acquired on a Zeiss LSM880 upright microscope with Airyscan. Post-acquisition analysis was performed using Zen software (Zen version 2015 black edition 2.3; Zeiss) or Fiji (version 1.49) software. Fluorescence intensities were calculated on > 10 cells from at least two independent experiments.

### Virus Preparation and infection

TBEV strain Hypr cDNA clone (U39292) and SP6 transcription in vitro have been described previously [38]. Briefly, sequence verified DNA plasmids encoding the relevant clone were linearized at the *Sma*I site (*Sma*I is an endonuclease enzyme isolated from strains of *serratia marcescens* and is part of the restriction modification system) downstream of the TBEV coding sequence and used as a template to produce full-length capped RNA using SP6 RNA polymerase (Promega). Each reaction (50 µL total volume) contained 1 µg of linearized DNA template and 40 units of SP6 RNA polymerase, incubated at 37 °C for 3 h. RNAs were purified using the SV total RNA isolation kit (Promega) and resuspended in RNase-free water (Invitrogen). RNA integrity was verified by agarose gel (1%) electrophoresis and quantified by spectrophotometry. Transfections were performed using Lipofectamine 3000 (Invitrogen). Briefly, 1 µg of SP6 transcribed RNA was complexed with transfection reagent in serum- and antibiotic-free culture medium for 15 min at room temperature. Complexes were added to porcine kidney (PS) cell monolayers for 24 to 96 h. All transfections were performed in triplicate. Infectious supernatants were collected at 24 to 72 h post infection (hpi). Aliquots of virus were diluted in serum-free RPMI 1640 and added to monolayers of PS cells for 1 h at 37 °C. Inoculum was aspirated, and plates were overlaid with RPMI 1640 supplemented with 2% FCS and 1% SeaPlaque Agarose (Cambrex) for 5 days at 37 °C for plaque formation. Monolayers were fixed in 4% paraformaldehyde and stained with 0.05% crystal violet. Plaques were counted and virus titres expressed as log_10_ PFU/mL. All virus work was performed in a BSL3 laboratory.

For TBEV growth kinetics, human motor neurons were infected at an MOI of 0.1 for 1 h. Infected cells were washed five times with PBS and incubated with fresh complete neuronal medium at 37 °C. Supernatants from infected cells were collected at 0, 24, 48 and 72 hpi and frozen at − 80 °C prior to analysis. Experiments were performed in triplicate. Titres of infectious virus at each time point were determined by plaque assay.

### ELISA analysis

Supernatants from mock and TBEV infected (MOI = 1) human motor neurons (4 × 10^5^ cell/mL) were collected at 24- and 96-hpi for metabolite analysis. S-Adenosylmethionine (SAM), Fructose-1,6-bisphosphatase 1 (FBP1) and Phosphoenolpyruvic acid (PEP) levels were determined using Human CEG414Ge (Cloud-Clone Corp), A313775 (Antibodies.com) and ab204713 (Abcam) ELISA Kits, respectively. The human IL-10 level was measured using the EHIL10 ELISA kit (Invitrogen). Levels were measured on a NanoQuant Infinite M200 plate reader (Tecan).

### Cytotoxicity assays

Neurons grown on 96-well plates were infected with TBEV (Hypr; MOI = 1) or treated with inhibitors (Sinefungin, MB05032 [0.0, 0.1, and 1.0 µM], NaF [0.0, 0.1, and 1.0 mM]) at various concentrations in triplicate for up to 96 h. Media was replenished with fresh media supplemented with inhibitors every 24 h. At the indicated time points, cells were trypsinised for 2 min (20 µl 0.25% trypsin; Gibco) at 37 °C. Cells were resuspended in 80 µl neuronal of differentiation media and mixed with 4% trypan blue solution (1:1 volume). The number of viable cells were counted using a haemocytometer.

### Inhibition of TBEV release with sinefungin, MB05032 and NaF

The following inhibitors were profiled: Sinefungin (Sigma) for S-Adenosylmethionine [SAM], NaF (Sigma) for Phosphoenolpyruvic acid (PEP), and MB06322 (SML3207, Sigma) for Fructose 1,6-bisphosphate (FBP1). Human motor neurons (hMNs) were grown to confluency on 96 well plates and treated with inhibitors for 24 h. Media was removed, and cells were infected with TBEV for 4 h at an MOI of 1 in media containing inhibitors. After 4 h, media was replaced with fresh media plus inhibitors. At 24, 48 and 72 hpi, media was collected for the assessment of extracellular viral titres determined using plaque assay. Data represent the mean ± standard deviation (SD) from three biologically independent samples. *P*-values were calculated using a two-way ANOVA followed by Bonferroni multiple comparison test.

### Glucose and G6P measurement

Extracellular glucose was measured in hMN culture supernatants using a glucose assay kit (MAK476, Merck), according to the manufacturer’s instructions. Intracellular glucose-6-phosphate (G6P) levels were measured using the G6P assay kit (MAK014, Merck) from cell lysates collected at 24 h post-infection in the presence or absence of the FBP-1 inhibitor, MB05032 (1 µM)]. Absorbances were read at 570 nm (glucose) or 450 nm (G6P) on a NanoQuant Infinite M200 plate reader (Tecan).

### Data Availability

All data are included in the article or in the supplementary information.

## Results

### Heterogeneity in disease outcomes of hospitalised TBEV-infected patients

**Fig. 1:**
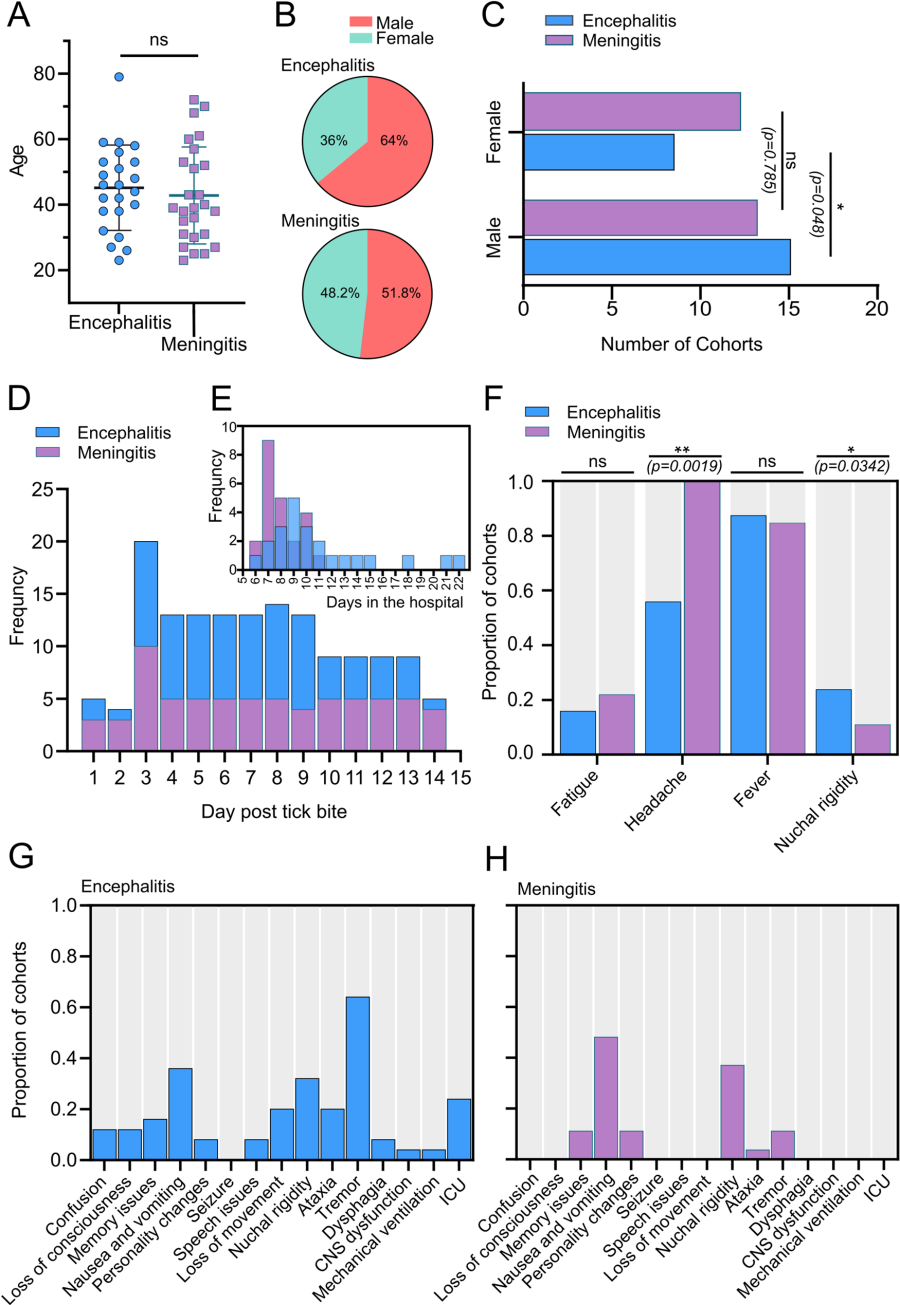
Demographics and reported symptoms of encephalitis (blue) and meningitis (purple) groups. Data were collected upon hospital admission with consent (*n* = 52 biologically independent patients). (**A**) No differences in biological age were observed across encephalitis and meningitis groups (ns, *p* = 0.675). (**B**) A pie chart shows the percentage of gender representation in each group, with females in green and males in orange. (**C**) Gender association with encephalitis (blue) and meningitis (purple). Statistical significance was determined using a two-proportion z-test (z = 1.98, *p = 0.048); ns, not significant. (**D**) Frequency and (**E**) peak hospitalisation days post-tick bite for the encephalitis and meningitis groups. (**F**) Non-neurological symptoms for patients in the encephalitis (blue) and meningitis (purple) groups. Proportion and peak severity of non-neurological symptoms experienced by infected patients since acquiring TBEV. Proportion and peak severity of neurological symptoms over the hospital administration period for patients in (**G**) encephalitis and (**H**) meningitis groups. Statistical significance was determined using a two-way ANOVA followed by Bonferroni multiple comparison test (*p < 0.05, **p < 0.01 ***p < 0.001, ****p < 0.0001); ns, not significant

Samples were obtained at the time of hospitalisation during the second phase of disease. The study groups were of comparable age (Fig. 1A). A higher proportion of males presented with encephalitis (64%, *p* = 0.0048), whereas no significant differences in gender were observed for meningitis (51.8%, *p* = 0.785) (Fig. 1B, C). All cohorts developed TBE following a TBEV-Hypr infected tick bite. A high frequency of hospital admissions was recorded 3 days after the tick bites for both meningitis and encephalitis groups (Fig. 1D). In the encephalitis group, 11 out of 25 patients noticed a tick bite approximately 3 to 5 days prior to hospitalisation, compared to 20 out of 27 in the meningitis group. The inpatient period was shorter for the meningitis group (mean number of days = 7) compared to the encephalitis group (mean of 9 days; Fig. 1E). All cohorts experienced four systemic symptoms: fatigue, headache, fever, and nuchal rigidity. Notably, the incidence of headaches was significantly higher in the meningitis group (*p* = 0.0019), while nuchal rigidity was more prevalent among encephalitis patients (*p* = 0.0342) (Fig. 1F). Neurological symptoms, including CNS dysfunction, mechanical ventilation, and dysphagia occurred in 1/6 encephalitis patients requiring treatment in the intensive care unit (ICU). Memory loss, nausea and vomiting, personality changes, nuchal rigidity, ataxia and tremor were common in both encephalitis (Fig. 1G) and meningitis groups (Fig. 1H). All patients admitted to the ICU were male and diagnosed with encephalitis (Supplementary Table S1). Those treated in the ICU exhibited the longest hospital admission period. Among the cohort, the longest hospitalisation period was 22 (*n* = 2 patients) days.

### Analysis of chemokine and cytokine levels in the CSF of TBE patients

**Fig. 2:**
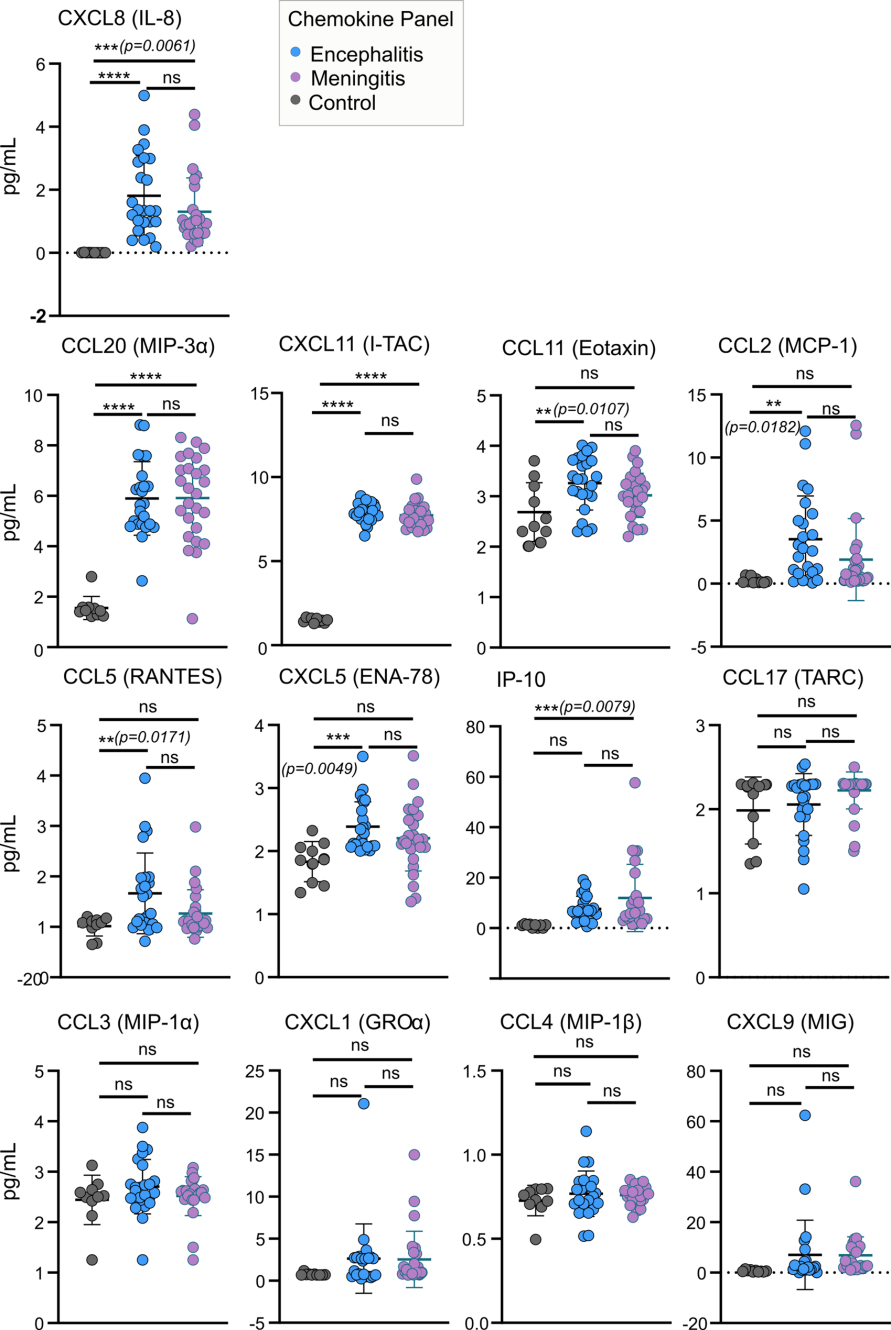
Human proinflammatory chemokine profiling in the cerebrospinal fluid of hospitalised TBEV patients and controls. Panels consisting of 13 human chemokines (IL-8, IP-10, CCL11, CCL17, CCL2, CCL5, CCL3, CXCL9, CXCL5, CCL20, CXCL1, CXCL11 and CCL4) were assayed using a multiplex bead-based system. Significance was determined using a one-way ANOVA followed by Bonferroni multiple comparison test (*p < 0.05, **p < 0.01 ***p < 0.001, ****p < 0.0001); ns, not significant. Data are the mean ± standard deviation of 3 technical repeats and 2 independent experiments

**Fig. 3:**
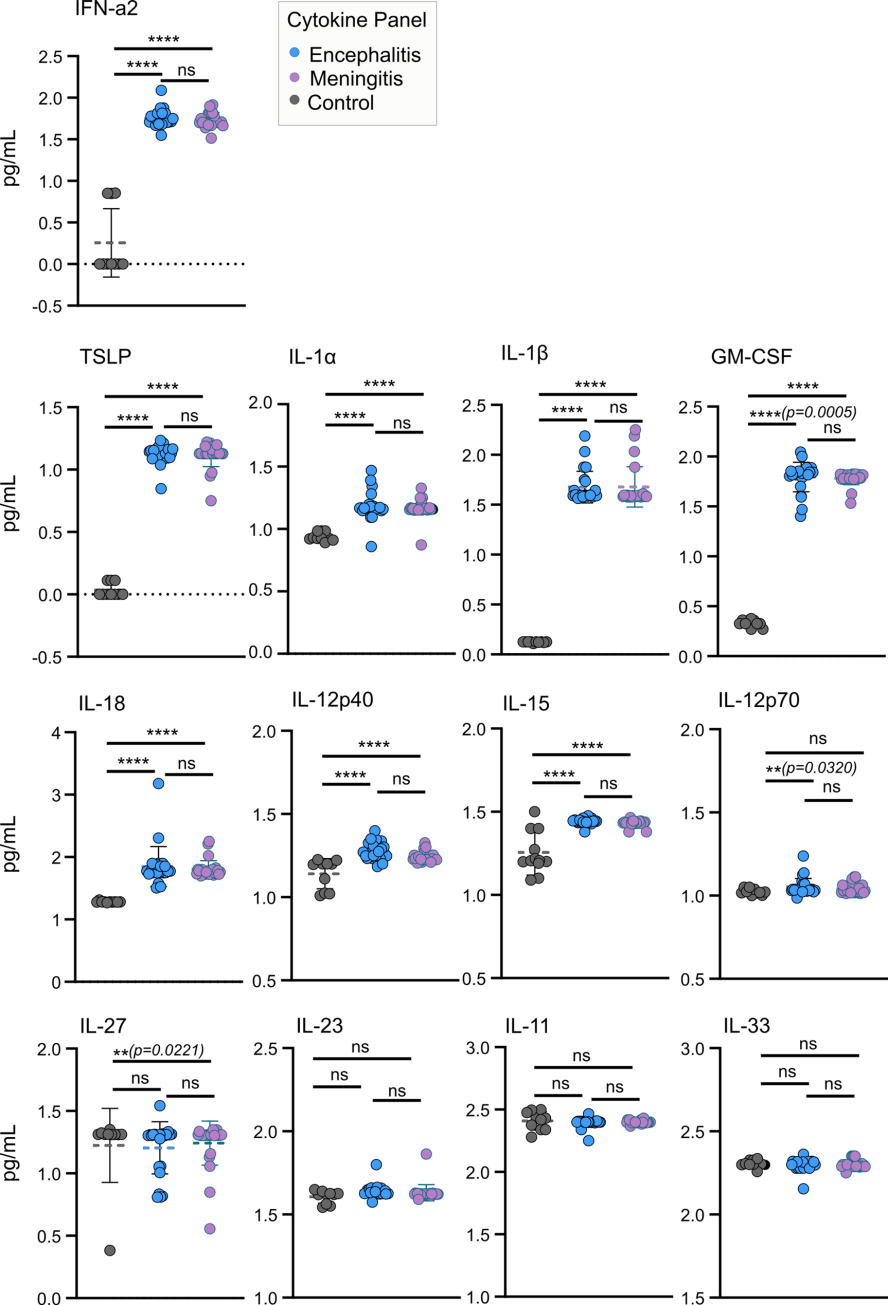
Cytokine profiling in the cerebrospinal fluid of hospitalised TBEV patients and controls. Panels consisting of 13 human cytokines (TSLP, IL-1α, IL-1β, GM-CSF, IFN-α2, IL-23, IL-12p40, IL-12p70, IL-15, IL-18, IL-11, IL-27, and IL-33) were tested using a multiplex bead-based assay. Significance was determined using a one-way ANOVA followed by Bonferroni multiple comparison test (*p < 0.05, **p < 0.01 ***p < 0.001, ****p < 0.0001); ns, not significant. Data are the mean ± standard deviation of 3 technical repeats and 2 independent experiments

To assay the correlation between disease phenotype and inflammatory responses, chemokines (Fig. 2) and cytokines (Fig. 3) were measured in the CSF samples from TBE patients and controls. Amongst the 13 proinflammatory chemokines (CXCL8 (IL-8), CXCL10 (IP-10), CCL11 (Eotaxin), CCL17 (TARC), CCL2 (MCP-1), CCL5 (RANTES), CCL3 (MIP-1α), CXCL9 (MIG), CXCL5 (ENA-78), CCL20 (MIP-3α), CXCL1 (GROα), CXCL11 (I-TAC), CCL4 (MIP-1β) tested (Fig. 2) a significantly higher levels of CXCL8 (*p* = 0.0061), CCL20 (*p* = < 0.0001) and CXCL11 (*p* = < 0.0001) were observed in both encephalitis and meningitis groups compared to controls. Significantly higher levels of CCL11 (*p* = 0.0107), CCL2 (*p* = 0.0182), CCL5 (*p* = 0.0171) and CXCL5 (*p* = 0.0049) were observed in encephalitis patients. IP-10 (*p* = 0.0079) levels in meningitis patients were elevated compared to controls. No significant differences were detected between meningitis and encephalitis groups.

We further measured the expression of 13 human cytokines (IFN-α2, TSLP, IL-1α, IL-1β, GM-CSF, IL-11, IL-12p40, IL-12p70, IL-15, IL-18, IL-23, IL-27, IL-33) in the CSF using a multiplex bead-based assay panel (Fig. 3). No variations in cytokine levels between meningitis and encephalitis groups were detected. Marked increases (*p* = ≥ 0.0005) were however, observed in IFN-α2, TSLP, IL-1α, IL-1β, GM-CSF, IL-18, IL-12p40, and IL-15 in both groups compared to controls. IL-12p70 (*p* = 0.0320) was also elevated in the encephalitis group and IL-27 (*p* = 0.0221) in the meningitis group compared to the control group. Together, this highlighted a broad and robust pro-inflammatory response in hospitalised TBE patients that was comparable between meningitis and encephalitis groups. In line with previous observations [42], we did not detect significant differences in the anti-inflammatory cytokine IL-10 (Supplementary Fig. S1) between the TBEV groups and controls, which remained at low levels compared to pro-inflammatory markers.

### Changes in the CSF metabolome in TBE patients are associated with neurological symptoms

**Fig. 4:**
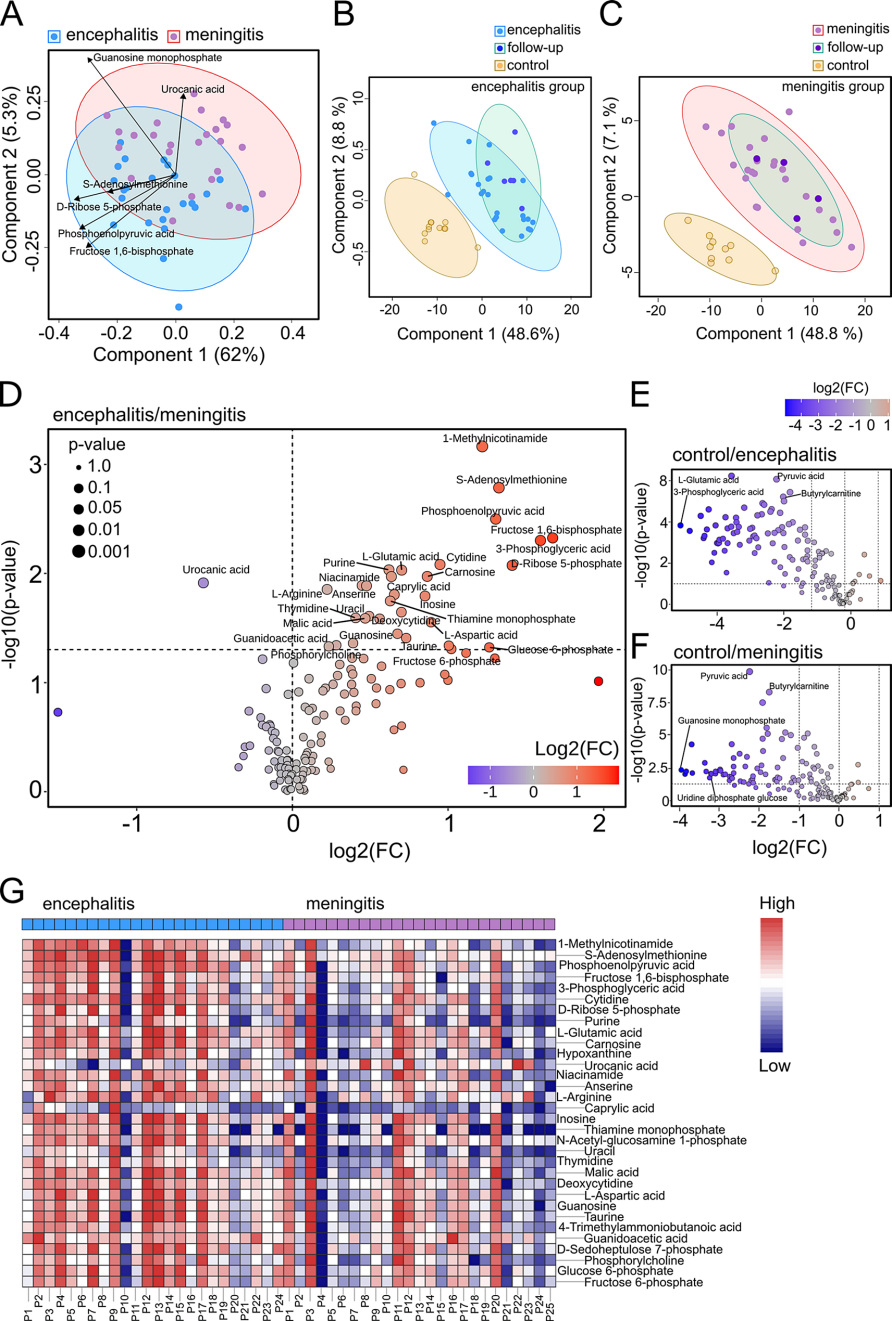
Metabolomic profiles in cerebrospinal fluid samples from control, encephalitis and meningitis TBEV patients. (**A**) 2D PLS-DA plots with 95% confidence ellipses showing the effects of encephalitis and meningitis on the CSF metabolome. The metabolites contributing to the separation of samples are labelled with arrows indicating their direction and magnitude. Cross-validated 2D PLS-DA score plot comparing TBEV patients with (**B**) encephalitis (with follow-up) and control, or TBEV patients with (**C**) meningitis, follow-up, and control groups on the CSF metabolome. Volcano plots comparing a two-fold change in concentration of CSF metabolites in (**D**) encephalitis vs. meningitis, or (**E**) control vs. encephalitis or (**F**) meningitis patients. Significantly altered metabolites are highlighted in red (increased) or blue (decreased). Data were compared using a two-sided Mann-Whitney U test followed by Benjamini-Hochberg multiple comparison test with FDR < 0.05 and fold-change > 1. (**G**) Heatmap showing CSF metabolites of significantly higher (red) or lower (blue) abundance in TBEV-patients with encephalitis vs. meningitis. Metabolite alterations are represented by colour intensity. Borders are colour-coded according to statistical significance (*p* < 0.05)

We next examined metabolic perturbations in the CSF associated with each TBE patient group using untargeted metabolomics (encephalitis, *n* = 24; meningitis, *n* = 25) and controls (*n* = 12). A single patient in the encephalitis group and two from the meningitis group were excluded from these analyses due to limited sampling. CSF metabolites were extracted and analysed using liquid chromatography-mass spectrometry (LC-MS), without significant systemic drift. A total of 151 metabolites were analysed. The levels 32 metabolites significantly differed (*p* = < 0.05) between encephalitis and meningitis patients (Supplementary Table S3). A total of 77 metabolites differed between control and encephalitis patients (Supplementary Table S4). A total of 61 metabolites differed between control and meningitis patients (Supplementary Table S5). A 2D PLS-DA scores plot, with 95% confidence ellipses, showed that the CSF metabolic profiles of patients with encephalitis and meningitis were distinct (Fig. 4A). Despite some clustering (overlapping regions), Components 1 and 2 accounted for 62% and 5.3% of the total variance, respectively. Key metabolites, including Guanosine monophosphate, S-Adenosylmethionine, Phosphoenolpyruvic acid, D-Ribose 5-phosphate, Fructose-1,6-bisphosphatase 1, and Urocanic acid, are highlighted by arrows to indicate their direction and magnitude of contribution to the separation of samples. PLS-DA analysis comparing the metabolic profiles of TBE patient groups with their respective follow-up revealed predominantly overlapping metabolite profiles, but a clear metabolic distinction from the control group (Fig. 4B-C).

A volcano plot (Fig. 4D) identified 32 metabolites showing 2-fold changes (FDR < 0.05), with 31 metabolites significantly increased (including S-Adenosylmethionine, Phosphoenolpyruvic acid, and Fructose-1,6-bisphosphatase 1) and 1 metabolite (Urocanic acid) significantly decreased in the encephalitis group compared to the meningitis group. Follow-up sample analysis revealed a 2-fold increase in the concentration of O-Phosphoethanolamine (O-PEA) (*p* = 0.004586) and a 2-fold decrease in the concentration of two metabolites (succinic acid, *p* = 0.030165; L-Proline, *p* = 0.033627) compared to the encephalitis group. Additionally, Pyruvic acid (*p* = 1.4685e-12) showed a 2-fold increase in concentration in follow-up samples compared to the meningitis group. Volcano plots further identified differing levels of metabolites in the encephalitis (Fig. 4E) (FDR < 0.05, 2-fold change: 76 downregulated and 1 upregulated [3-Methylhistidine]) and meningitis (Fig. 4F) (FDR < 0.05, 2-fold change: 61 upregulated) groups compared to controls. Further ranking of significantly (*p* = < 0.05) altered metabolites in TBEV patients showed an elevated abundance of metabolic products in the encephalitis group compared to the meningitis group (Fig. 4G).

**Fig. 5:**
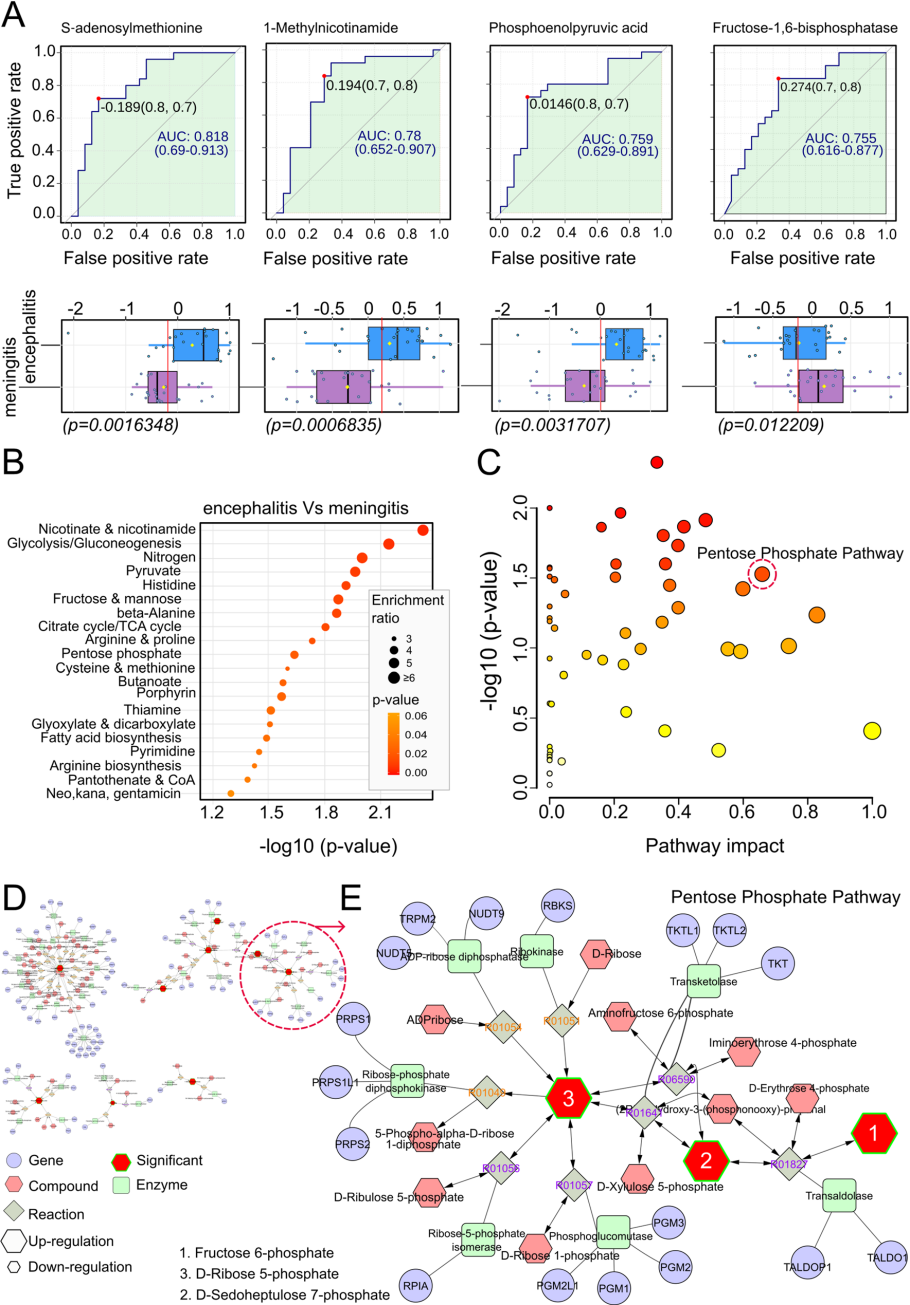
Changes in the cerebrospinal fluid metabolomes in encephalitis and meningitis TBEV. (**A**) Area under the curve (AUC) in the top panel and box plot representation in the lower panel, showing cut-off values of 4 CSF biomarkers with significant up-regulation (S-Adenosylmethionine: AUC = 0.818, *p* = 0.0016348; 1-Methylnicotinamide: AUC = 0.78, *p* = 0.0006835; Phosphoenolpyruvic acid: AUC = 0.759, *p* = 0.0031707; Fructose 1,6-bisphosphate: AUC = 0.755, *p* = 0.012209) identified in encephalitis vs. meningitis. Statistical significance was determined using a one-way ANOVA followed by Bonferroni multiple comparison test (*p < 0.05, **p < 0.01 ***p < 0.001, ****p < 0.0001); ns, not significant. (**B**) KEGG metabolic pathway enrichment analysis and (**C**) scatter plot of pathway impact versus pathway significance, where each node represents a significant metabolic pathway contributing to the differences between encephalitis and meningitis TBEV-patient groups. The white to red node colour scale indicates the *p*-value, and node size reflects the pathway impact score. Red circles indicate the most highly affected metabolic pathways in TBEV patients, with the highest number of metabolite hits (9) from the pathway analysis (Impact score = 0.65877). (**D**) Network diagrams of metabolites in the CSF from encephalitis and meningitis patients. (**E**) Magnification of a new cluster of the PPP visualized from (**D**) in Cytoscape

To identify candidate metabolites with the greatest power to discriminate amongst TBEV patient groups, we selected ‘discriminative metabolites’ that showed an area under the curve (AUC) ≥ 0.71 for a given comparison between encephalitis and meningitis groups. Biomarker analysis (Fig. 5A) showed 11 metabolites that were identified as discriminative (AUC ≥ 0.71; Supplementary Table S6), comprising 4 metabolic pathway intermediates (PEP, FBP-1, 3-Phosphoglyceric acid [3-PGA], D-Ribose 5-phosphate [R5P]), 3 nucleosides/nucleotides (Cytidine, Thymidine, Hypoxanthine), 1 amino acid (L-Arginine), vitamins and derivatives (1-Methylnicotinamide), coenzymes (S-Adenosylmethionine [SAM]) and fatty acids (Caprylic acid).

Enrichment analysis revealed differentially abundant metabolic pathways between encephalitis and meningitis TBEV patients (Fig. 5B). KEGG pathway analysis identified significant alterations in 19 metabolic pathways (Fig. 5C, *p* < 0.05), with the pentose phosphate pathway (PPP) the most significantly affected, with changes in 9 metabolites (Impact score = 0.65877) (Fig. 5C). To visually integrate the network with PPP in TBE patients with CNS disease, a correlation-based network analysis was performed (Fig. 5D, E). This underscored the complexity and extensive metabolic interactions in the CSF during the second phase of TBEV infection.

### TBEV infection in human motor neurons confirms elevated levels of S- Adenosylmethionine, Fructose-1,6-bisphosphatase 1 and Phosphoenolpyruvic acid

**Fig. 6:**
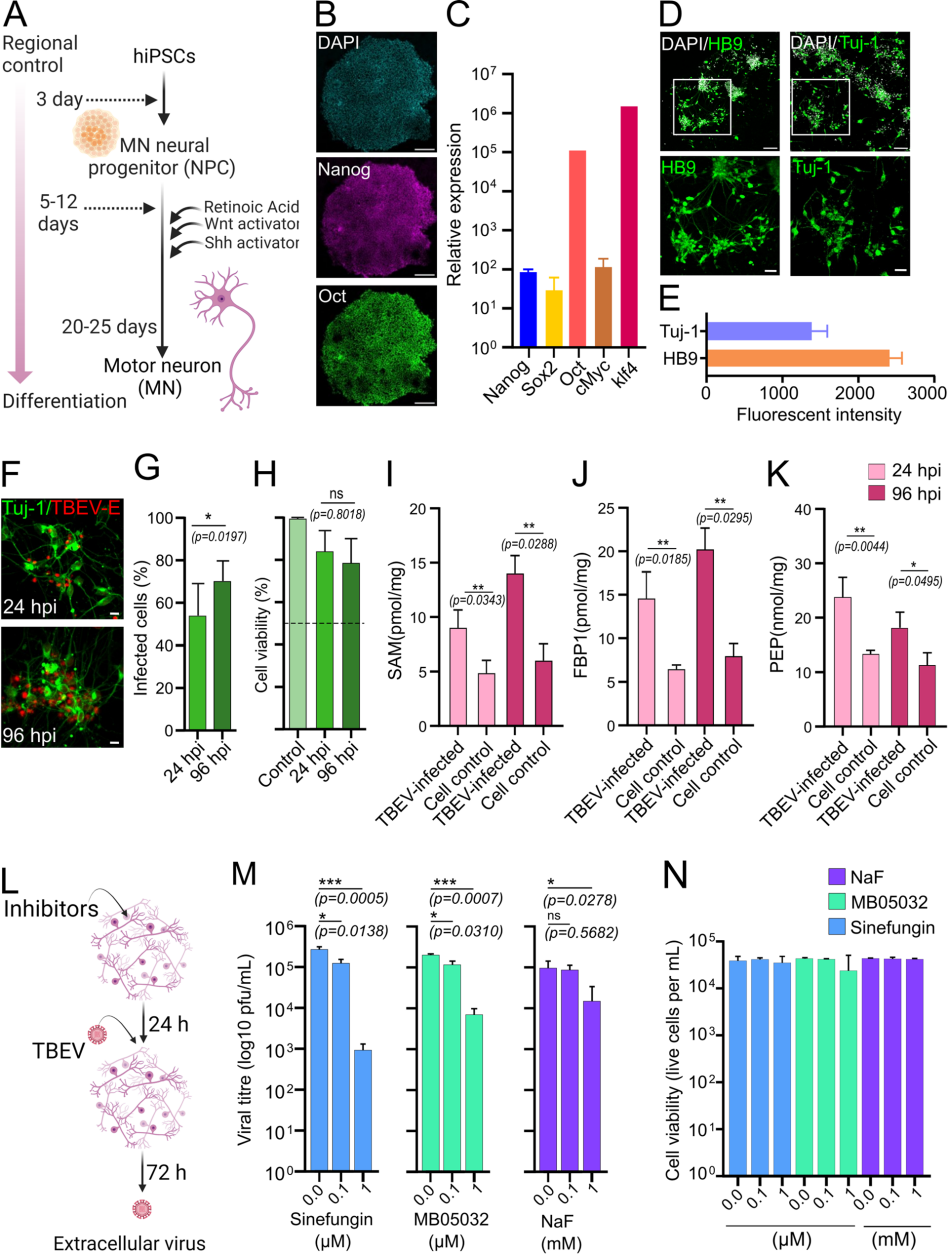
Identified metabolites impact TBEV infection in human motor neurons (hMNs). (**A**) Schematic showing neuronal differentiation achieved from human iPSCs with a combination of CHIR99021, an activator of Wnt signaling and purmorphamine a SHH activator. (**B**) Validation of pluripotency by immunostaining of Nanog and Oct. Cells were counterstained with DAPI. (**C**) qRT-PCR analysis of Nango, Sox2, Oct, cMyc and klf4 expression in human iPSCs. (**D**) Validation of hMNs generated from (**A-C**). Expression of HB9 and Tuj-1 quantified by immunofluorescence. (**E**) Fluorescent intensities are shown for Tuj-1 and HB9 from four technical repeats in two independent experiments. (**F**) Confirmation of TBEV infection at an MOI of 0.1 in hMNs. Cells were stained with anti-E proteins (red) and Tuj-1 (green) at 24- and 96-h post-infection (hpi). (**G**) Quantification of the % of infected cells and (**H**) cell viability studies. (**I**) Bar graphs of soluble SAM, (**J**) FBP1 and (**K**) PEP levels in infected neurones at 24- and 96-hpi. Infection of hMNs with TBEV at 1 MOI, followed by ELISA. (**L**) Schematic of the assay design. (**M**) hMNs were treated with increasing concentrations of Sinefungin, MB05032 [0.0, 0.1, and 1.0 µM], NaF [0.0, 0.1, and 1.0 mM]) for 24 h and infected with TBEV at an MOI of 0.1 PFU, plaque-forming units. Viral titres at 72 hpi. were determined via plaque assay. (**N**) For viability studies, hMNs were treated with inhibitors and infected with TBEV at an MOI of 1 PFU. Statistical significance was determined using a one-way ANOVA followed by Bonferroni multiple comparison test (*p < 0.05, **p < 0.01 ***p < 0.001, ****p < 0.0001); ns, not significant. Data are the mean ± standard deviation of 3 technical repeats in 3 independent experiments. A and L created using BioRender.com

For confirmation of the differences observed in CSF metabolites across the neurological-TBE patient groups, the levels of S-Adenosylmethionine (SAM), Fructose-1,6-bisphosphatase 1 (FBP1) and Phosphoenolpyruvic acid (PEP) were profiled in human iPSC-derived motor neurons (hMN) (Fig. 6A) infected with TBEV strain Hypr. Hypr belongs to the European subtype of TBEV and was isolated from a human patient with severe TBE in the Czech Republic. iPSCs were validated through elevated expression of Nango, Sox2, Oct, cMyc and klf4 (Fig. 6B, C) and HB9 and Tuj-1 in neurons (Fig. 6D, E) detected through immunostaining and qPCR. These confirmed pluripotency and neuronal identity, respectively. Virus permissibility in hMNs was confirmed by immunofluorescent staining for TBEV-E protein, used as a marker of infection (Fig. 6F). The percentage of infected cells was significantly (*p* = 0.0197) higher at 96 hpi compared to 24 hpi (Fig. 6G), with no significant cytotoxicity observed at any time point (Fig. 6H), confirming the suitability of the hMN model for TBEV investigation. The levels of SAM (Fig. 6I) and FBP1 (Fig. 6J) significantly increased in hMNs 24 hpi and continued up to 96 hpi. In contrast, higher levels of PEP were detected at early time points (24 hpi) (Fig. 6K). No such changes were observed in uninfected neurons.

To evaluate the impact of SAM, FBP1 and PEP on TBEV replication and release, neurons were treated with increasing concentrations of Sinefungin, MB06322 and NaF, respectively, for 24 h prior to infection with TBEV (Hypr) (Fig. 6L, M). Treatment with Sinefungin and MB06322 resulted in a concentration dependent reduction in TBEV titres at 72 hpi (Fig. 6M). A < 0.5 log reduction in TBEV was observed following NaF treatment at 72 hpi, which was most effective at early time points, exhibiting a log reduction of virus release at 24 hpi (Fig. 6M, and Supplementary Fig. S2B). Treatment with 1 µM of SAM, FBP1, and PEP inhibitors led to a 20-200-fold reduction in TBEV at all time points, highlighting their ability to supress TBEV replication (Fig. 6M and Supplementary Fig. S2C, D). The reduction was not a result of cytotoxicity, as treatment with inhibitory concentrations of Sinefungin, MB06322 and NaF did not affect hMN viability (Fig. 6N) but did lead to lower levels of downstream metabolites (Supplementary Fig. S1A). TBEV infection also significantly altered cellular glucose metabolism. Compared to uninfected controls, TBEV-infected cells exhibited a marked reduction in extracellular glucose concentrations (*P* = 0.0065), indicating increased glucose uptake (Supplementary Fig. S1E, left). This was accompanied by a significant increase in intracellular glucose-6-phosphate (G6P) levels (*P* = 0.00444), suggestive of elevated glycolytic flux (Supplementary Fig. S1E, right). Treatment with the FBP1 inhibitor MB05032 (1 µM) did not significantly affect extracellular glucose (*P* = 0.1874) but led to increased intracellular G6P levels (*P* = 0.0335), confirming a TBEV mediated shift in metabolism (Supplementary Fig. S1F). Collectively, these findings confirmed the power of our metabolic analysis and revealed key metabolites as biomarkers for TBEV-associated neuroinflammatory disease.

## Discussion

Deeper knowledge of TBEV neuropathogenesis in the most relevant models of virus infection are needed to identify strategies for improved diagnosis and patient management. To achieve this, we performed the most comprehensive analysis of cerebrospinal fluid (CSF) from TBEV patient’s to-date, focussing on patients who progressed to encephalitis or meningitis. Neurological symptoms and disease associated with encephalitis are typically more severe and require urgent intervention. By stratifying our analysis according to these outcomes, we profiled the induction of host markers as signatures to differentiate the severity of TBE cases and ultimately patient management.

In agreement with previous studies [43–45], we observed increased levels of numerous chemokines and cytokines in the CSF of patients infected with TBEV, correlating with the inflammation observed during the second stage of the disease. The elevated presence of CXCL8, CCL20, and CXCL11 (Fig. 2) in the CSF of both study groups were indicative of the recruitment of immune cells, including neutrophils, T-cells, and dendritic cells. Whereas pro-inflammatory cytokines such as IFN-α2, TSLP, IL-12p40, IL-18, and IL-15 (Fig. 3) demonstrate the maturation and activation of immune cells (T cells, dendritic cells, and NK cells) and a robust antiviral response. The expression of IL-1α and IL-1β in the CSF correlated with symptoms (inflammation and fever) linked to both encephalitis and meningitis groups [46]. Additionally, marked elevation of several immune markers (CCL11, CCL2, CCL5, CXCL5, and IL-12p70) in the CSF of encephalitis patients may explain the more severe course of infection and poorer outcomes observed (highest number of neurological symptoms and ICU admissions, Fig. 1G). Despite this, the levels of these immune markers did not differ significantly between meningitis and encephalitis groups, indicating a potentially shared mechanism of immune induction. Increased levels of CCL4 (Japanese encephalitis virus, West Nile virus [WNV]) [47, 48] and IL-33 (WNV) [49] have been linked to neuroinflammation following virus infection, but no changes in their levels were observed in TBEV-infected CSF samples compared to controls. Although the underlying mechanism(s) of immune induction remain unclear, the identified panel of infiltrates holds potential as a diagnostic signature for the rapid identification of TBEV infection. These immune markers, however, cannot distinguish between the different neuroinflammation outcomes of TBEV infection.

Metabolomics of the CSF has emerged as a key tool to understand the pathogenesis of neurotropic viral infections. ZIKA virus (ZIKV) has been shown to induce lipid droplet formation in neuronal cells, facilitating viral replication whilst contributing to neuroinflammation [50]. WNV infection prompts a glycolytic shift in the CNS through the upregulation of specific hexokinase isoforms and through the downregulation of mitochondrial respiratory enzymes. These changes act to support virus replication whilst exacerbating inflammatory responses [51]. For ZIKV and WNV, the interferon-induced enzyme cholesterol-25-hydroxylase (CH25H) restricts virus infection through altering lipid composition [52]. Complementary to our findings, CSF metabolomic analyses in patients with TBE revealed four metabolites (kynurenic acid, 5-hydroxyindole-3-acetic acid, DL-tryptophan, indole-3-acrylic acid) associated with a fatal outcome [53], though assessment in neuronal models of TBEV infection was not performed. The key finding of this study was the identification of distinct differences in CSF metabolites between meningitis and encephalitis patients (Fig. 4). The most prominent fold increases in encephalitis compared to meningitis patients were SAM (2.5-fold), FBP1 (3.2-fold), 1-Methylnicotinamide (MNA) (2.3-fold) and PEP (2.5-fold). However, CSF urocanic acid levels were ∼ 0.7-fold lower in the encephalitis group compared to the meningitis group. SAM is a critical methyl donor involved in epigenetic regulation and neuroinflammatory responses [54]. Changes in FBP1 and PEP are suggestive of altered glucose metabolism, which may reflect metabolic reprogramming under neuroinflammatory stress. MNA, derived from nicotinamide, has been linked to neuroprotection, but is indicative of altered NAD + metabolism, a hallmark of viral CNS infections [55–57]. In contrast, decreased urocanic acid, a histidine metabolite with an immunomodulatory role, may reflect impaired immune homeostasis [58]. Together, these changes likely contribute to the more severe neurological manifestations observed during TBEV associated encephalitis.

To-date, only a limited number of reports describe iPSC-derived neuronal models for TBEV studies [59]. Here, we developed hMNs assays (Fig. 6), a specialised class of neuron responsible for the transmission of efferent signals from the CNS to peripheral muscles, enabling voluntary movement and neuromuscular coordination [60]. Given their pivotal role in motor function, the frequently represent sites of infection for neurotropic viruses associated with motor deficits. In the context of TBEV infection (frequently associated with acute paresis and paralysis), hMNs represent a physiological model for TBEV associated neuropathology. In this study, we characterised hMNs derived from iPSCs and identified a specific subset that were directly susceptible to TBEV infection. Studies in primary hMNs infected with TBEV confirmed direct virus-induced alterations in each of the identified metabolites (Fig. 6 and Supplementary Fig. S2). Pharmacological inhibition of SAM and FBP1 also reduced virus infection at late lifecycle stages. PEP inhibition prevented early virus release. Collectively, this revealed for the first time how CNS metabolites fundamentally differ between meningitis and encephalitis cases and influence the TBEV lifecycle. SAM and FBP1 have been reported to influence the lifecycles of ZIKV [61], Epstein-Barr virus [62] and hepatitis B virus [63]. During DENV infection, the levels of PEP similarly increased during the early phase of virus infection [64]. The PPP is associated with chronic neuroinflammation in severe COVID-19 cases [65], also identified as an impact pathway among meningitis and encephalitis groups. The PPP operates during oxidative and non-oxidative phases; the non-oxidative phase is responsible for the interconversion of sugar phosphates, the intermediates of which feed into glycolysis and gluconeogenesis to produce ribose 5-phosphate (R5P) and nicotinamide adenine dinucleotide phosphate (NADPH) [66]. FBP1 through its role in gluconeogenesis, indirectly influences the flux of intermediates including fructose-6-phosphate and glyceraldehyde-3-phosphate [67]. TBEV, like other orthoflaviviruses [50, 51, 64], is therefore likely to reprogram host cell metabolism to support virus replication and supress immune responses. NADPH and R5P, both products of the PPP, play distinct yet complementary roles in these processes [66]. NADPH is essential for the maintenance of redox homeostasis and lipid biosynthesis, both critical for virus replication and host cell survival. NAPDH also contributes to the generation of reactive oxygen species (ROS) via NADPH oxidases, which can amplify neuroinflammation. R5P is a crucial precursor for nucleotide biosynthesis, required to support RNA genome replication. Our metabolomic data revealed an upregulation of intermediates that would occur following PPP activation, supporting the hypothesis that TBEV exploits this pathway.

We further demonstrated that TBEV infection reprograms host glucose metabolism, as evidenced by increased glucose consumption and G6P accumulation (Supplementary Fig. S2E-F). This metabolic shift suggests an enhanced glycolytic flux following TBEV infection. Notably, treatment with the FBP1 inhibitor MB05032, which blocks the rate-limiting step of gluconeogenesis, did not alter extracellular glucose levels but led to a further increase in intracellular G6P. These results are consistent with a TBEV-driven reprogramming of glucose metabolism toward glycolysis, likely to meet the heightened energetic and biosynthetic demands of viral replication. Similar metabolic rewiring has been observed in cells infected with other orthoflaviviruses, including DENV, ZIKV, and WNV [51, 64], all of which increase glucose uptake and glycolytic activity to facilitate viral replication. Our findings suggest that TBEV employs a comparable strategy, reinforcing the notion that glycolytic metabolites may serve as conserved metabolic biomarkers of orthoflavivirus infection.

The study observed significant metabolite changes in follow-up samples compared to those taken at the time of hospitalisation, reflecting a complex interplay of metabolic pathways during the prolonged or recovery phase from neuroinflammation. These included increased levels of O-PEA, a metabolite linked to phospholipid metabolism and structurally similar to the neurotransmitter GABA [68], potentially influencing neurotransmission during the recovery from encephalitis. Conversely, a decrease in succinic acid, a key intermediate in the citric acid cycle (Krebs cycle), was observed, suggestive of a shift in energy metabolism, possibly due to reduced cellular respiration and/or altered mitochondrial function [69]. L-Proline, which plays a role in protein synthesis and cellular stress responses, was also of lower levels. Pyruvic acid, a key intermediate in glycolysis and the citric acid cycle [66], was higher in follow-up samples of meningitis patients. This increase may reflect enhanced glycolytic activity to meet increased energy demands during prolonged infection. Similar metabolic alterations have been observed in patients with severe COVID-19 [70]. These findings underscore the importance of key metabolic adaptations during prolonged or recovery phases of TBEV-induced neuroinflammation.

Analysis of the cohort demographics revealed other important information pertaining to TBEV infection (Fig. 1). Males showed higher susceptibility to severe encephalitis compared to females, consistent with the notion that genetic (e.g. ApoE gene) and metabolic factors exhibit sex-specific differences that influence the susceptibility to neuroinflammatory disease [71]. Male microglia (the primary immune cells in the brain) also tend to be more reactive and produce higher levels of pro-inflammatory cytokines compared to females, leading to more pronounced neuroinflammation [72]. Future studies will aim to further define these relationships and their role during TBEV infection.

### Limitations and future perspectives

The present study has several limitations. Firstly, we assessed patients who acquired TBEV infection exclusively via tick bites, excluding other routes of infection such as the alimentary route. Future studies should expand our findings to alternative routes of virus transmission. Secondly, this study was conducted in a single region, which may limit the generalisability of our results. Expanding the research to include patients from diverse geographical areas would further validate our findings. Finally, we included a relatively small number of longitudinal samples due to the invasive nature of lumbar puncture, which restricts frequent CSF collection. Future studies should address this limitation by incorporating larger sample sizes to track dynamic changes in metabolic profiles across different stages of infection, including asymptomatic phases and the initial non-CNS-TBEV infection phase. Broadening the investigation of identified metabolites to serum and urine samples could also provide diagnostic value and enhance our understanding of the dynamics of the metabolic changes identified.

## Conclusions

We provide the first evidence of the effects of TBEV infection on CNS metabolism using human CSF samples. Notably, we identified a significantly altered metabolic signature of 32-metabolites that defined the clinical presentation of TBEV-infection and progression to encephalitis or meningitis. We present evidence of significant metabolic changes during prolonged neuroinflammation induced by TBEV, highlighting associated fold-changes in four key metabolites. The metabolic biomarkers identified offer a roadmap to identify individuals at risk of severe TBEV-associated disease.

## Electronic supplementary material

Below is the link to the electronic supplementary material.

Supplementary Material 1

Supplementary Material 2

Supplementary Material 3

Supplementary Material 4

Supplementary Material 5

Supplementary Material 6

Supplementary Material 7

Supplementary Material 8

Supplementary Material 9

Supplementary Material 10

## Data Availability

All data are included in the article or in the supplementary information.
