# Supplementary material for: Changes in metabolite profiles in the cerebrospinal fluid and in human neuronal cells upon tick-borne encephalitis virus infection
Source: J Neuroinflammation. 2025 Jun 14;22:157. doi: 10.1186/s12974-025-03478-4 (PMC12166563; doi:10.1186/s12974-025-03478-4)
Supplement: Supplementary file 1 — Supplementary Material 1 [file 12974_2025_3478_MOESM1_ESM.docx]

| **Supplementary Table S1.** Clinical data from TBEV-infected encephalitis donors. |  |
| --- | --- |
|  |  |

| **ID** | **Year of Diagnosis** | **Age at Diagnosis** | **Sex** | **Number of days in hospital** | **Other symptoms** | **Notes** |
| --- | --- | --- | --- | --- | --- | --- |
| 1 | 2023 | 30 | Male | 10 | Tremor, Fever, Headache, Fatigue, Loss of consciousness, Confusion, Personality change |  |
| 2 | 2023 | 32 | Female | 8 | Nuchal rigidity, Tremor, Nausea, Fever |  |
| 3 | 2022 | 51 | Male | 13 | Nuchal rigidity, Tremor, Ataxia, Nausea, Fever, Neck stiffness | Treated in the Intensive care unit (ICU) |
| 4 | 2020 | 79 | Female | 9 | Tremor, Headache, Fever, Neck stiffness, Weakness of the moment/muscle |  |
| 5 | 2020 | 59 | Male | 6 | Headache, Fever, Memory loss |  |
| 6 | 2022 | 48 | Female | 8 | Headache, Fever, Memory loss |  |
| 7 | 2023 | 40 | Male | 13 | Nuchal rigidity, Headache, Fever, Neck stiffness | Follow-up (4-month) |
| 8 | 2022 | 46 | Male | 18 | Nuchal rigidity, Tremor, Dysphagia, Headache, Fever, Neck stiffness, Memory loss, Nausea | Treated in the Intensive care unit (ICU)  Follow-up (4-month) |
| 9 | 2020 | 53 | Male | 15 | Tremor, Headache, Fever | Treated in the Intensive care unit (ICU) |
| 10 | 2023 | 42 | Female | 10 | Nuchal rigidity, Tremor, Headache | Follow-up (4-month) |
| 11 | 2020 | 38 | Male | 9 | Tremor, Headache, Fever, Confusion, Loss of consciousness, Nausea, Speech loss |  |
| 12 | 2020 | 56 | Female | 10 | Tremor, Fever, Headache, Fatigue |  |
| 13 | 2021 | 44 | Female | 9 | Tremor, Fever, Headache, Nausea |  |
| 14 | 2021 | 46 | Male | 7 | Tremor |  |
| 15 | 2021 | 53 | Female | 10 | Tremor, Ataxia |  |
| 16 | 2021 | 59 | Male | 13 | Nuchal rigidity, Tremor, Fever, Headache, Nausea |  |
| 17 | 2021 | 49 | Female | 11 | Tremor, Ataxia, Fever, Headache, Fatigue, Weakness of the moment/muscle |  |
| 18 | 2021 | 26 | Male | not known | Nuchal rigidity, Tremor, Fever, Headache, Confusion, Loss of consciousness, Memory loss, Speech loss, Weakness of the moment/muscle | Treated in the Intensive care unit (ICU) |
| 19 | 2022 | 38 | Male | 9 | Nuchal rigidity, Tremor, Ataxia, Fever, Headache |  |
| 20 | 2022 | 42 | Male | 14 | Fever, Nausea, Weakness of the moment/muscle |  |
| 21 | 2022 | 58 | Male | 22 | Severe diffuse of central nervous system (CNS) dysfunction, Mechanical ventilation, Dysphagia, Fever, Headache | Treated in the Intensive care unit (ICU)  Follow-up (4-month). |
| 22 | 2022 | 23 | Female | 8 | Fever, Neck stiffness, Nausea | Follow-up (4-month) |
| 23 | 2023 | 27 | Male | 8 | Tremor, Fever, Headache, Personality change, Neck stiffness |  |
| 24 | 2021 | 37 | Male | not known | Fever, Headache, Fatigue |  |
| 25 | 2022 | 49 | Male | 22 | Fever, Headache, Tremor, Weakness of the moment/muscle | Treated in the Intensive care unit (ICU)  Follow-up (4-month) |
