# Supplementary material for: Changes in metabolite profiles in the cerebrospinal fluid and in human neuronal cells upon tick-borne encephalitis virus infection
Source: J Neuroinflammation. 2025 Jun 14;22:157. doi: 10.1186/s12974-025-03478-4 (PMC12166563; doi:10.1186/s12974-025-03478-4)
Supplement: Supplementary file 2 — Supplementary Material 2 [file 12974_2025_3478_MOESM2_ESM.docx]

**Supplementary Table S2.** Clinical data from TBEV-infected meningitis donors.

| **ID** | **Year of Diagnosis** | **Age at Diagnosis** | **Sex** | **Number of days in hospital** | **Other symptoms** | **Notes** |
| --- | --- | --- | --- | --- | --- | --- |
| 1 | 2023 | 27 | Male | 8 | Tremor, Fever  Headache, Nausea, Memory loss |  |
| 2 | 2022 | 52 | Female | 8 | Fever, Headache |  |
| 3 | 2020 | 25 | Female | 8 | Nuchal rigidity, Fever, Headache, Fatigue |  |
| 4 | 2022 | 61 | Male | 7 | Fever, Headache |  |
| 5 | 2022 | 70 | Male | 6 | Headache, Nausea |  |
| 6 | 2020 | 60 | Male | 7 | Fever, Headache, Nausea |  |
| 7 | 2022 | 72 | Male | 10 | Fever, Headache |  |
| 8 | 2023 | 53 | Male | 9 | Headache, Fatigue | Follow-up (4-month) |
| 9 | 2023 | 31 | Female | 7 | Headache | Follow-up (4-month) |
| 10 | 2021 | 25 | Male | 10 | Nuchal rigidity, Tremor, Fever, Headache, Nausea, Fatigue |  |
| 11 | 2020 | 31 | Male | not known | Fever, Headache, Nausea, Fatigue, Personality change |  |
| 12 | 2020 | 57 | Female | 7 | Nuchal rigidity, Fever, Headache, Nausea, Personality change |  |
| 13 | 2021 | 68 | Female | 8 | Fever, Headache |  |
| 14 | 2021 | 23 | Female | 9 | Nuchal rigidity, Fever, Headache, Nausea |  |
| 15 | 2021 | 40 | Female | 9 | Ataxia, Nuchal rigidity, Fever, Headache |  |
| 16 | 2021 | 35 | Male | 10 | Nuchal rigidity, Fever, Headache, Fatigue |  |
| 17 | 2021 | 36 | Male | 7 | Fever, Headache Nausea, Memory loss |  |
| 18 | 2022 | 38 | Male | 7 | Nuchal rigidity, Fever, Headache, Nausea, Personality change |  |
| 19 | 2022 | 43 | Male | 11 | Fever, Headache |  |
| 20 | 2023 | 38 | Female | 7 | Fever, Headache, Fatigue, Nausea, Memory loss |  |
| 21 | 2023 | 51 | Female | 7 | Nuchal rigidity, Fever, Headache, Nausea |  |
| 22 | 2023 | 30 | Female | 12 | Fever, Headache | Follow-up (4-month) |
| 23 | 2023 | 27 | Female | not known | Fever, Headache |  |
| 24 | 2023 | 39 | Male | 8 | Tremor, Fever, Headache, Nausea |  |
| 25 | 2023 | 43 | Male | 8 | Nuchal rigidity, Fever, Headache, Neck stiffness, Nausea |  |
| 26 | 2023 | 39 | Female | 9 | Nuchal rigidity, Fever, Headache |  |
| 27 | 2023 | 19 | Female | 10 | Fever, Headache, Nausea | Follow-up (4-month) |
