# Supplementary material for: Changes in metabolite profiles in the cerebrospinal fluid and in human neuronal cells upon tick-borne encephalitis virus infection
Source: J Neuroinflammation. 2025 Jun 14;22:157. doi: 10.1186/s12974-025-03478-4 (PMC12166563; doi:10.1186/s12974-025-03478-4)
Supplement: Supplementary file 3 — Supplementary Material 3 [file 12974_2025_3478_MOESM3_ESM.docx]

**Supplementary Table S3.** Significantly differed metabolites between encephalitis and meningitis.

| **Name** | **Fold Change (FC)** | **p-value** |
| --- | --- | --- |
| 1-Methylnicotinamide | 2.3288 | 0.000684 |
| S-Adenosylmethionine | 2.5082 | 0.001635 |
| Phosphoenolpyruvic acid | 2.4727 | 0.003171 |
| Fructose 1,6-bisphosphate | 3.1866 | 0.004724 |
| 3-Phosphoglyceric acid | 3.0179 | 0.004986 |
| Cytidine | 1.9293 | 0.008292 |
| D-Ribose 5-phosphate | 2.6601 | 0.008446 |
| Purine | 1.5372 | 0.009274 |
| L-Glutamic acid | 1.6267 | 0.009333 |
| Carnosine | 1.8229 | 0.010659 |
| Hypoxanthine | 1.5539 | 0.010689 |
| Urocanic acid | 0.6727 | 0.012209 |
| Niacinamide | 1.3639 | 0.0129 |
| Anserine | 1.391 | 0.012994 |
| L-Arginine | 1.1671 | 0.014023 |
| Caprylic acid | 1.5745 | 0.015646 |
| Inosine | 1.8046 | 0.016117 |
| Thiamine monophosphate | 1.5458 | 0.0179 |
| N-Acetyl-glucosamine 1-phosphate | 1.6247 | 0.022663 |
| Uracil | 1.4071 | 0.024683 |
| Thymidine | 1.3252 | 0.025621 |
| Malic acid | 1.3827 | 0.025841 |
| Deoxycytidine | 1.475 | 0.026088 |
| L-Aspartic acid | 1.8539 | 0.028036 |
| Guanosine | 1.5965 | 0.035794 |
| Taurine | 1.6597 | 0.039169 |
| 4-Trimethylammoniobutanoic acid | 1.31 | 0.043691 |
| Guanidoacetic acid | 1.216 | 0.045608 |
| D-Sedoheptulose 7-phosphate | 2.0065 | 0.045956 |
| Phosphorylcholine | 1.1759 | 0.046707 |
| Glucose 6-phosphate | 2.4025 | 0.047701 |
| Fructose 6-phosphate | 2.029 | 0.049405 |
