# Supplementary material for: Changes in metabolite profiles in the cerebrospinal fluid and in human neuronal cells upon tick-borne encephalitis virus infection
Source: J Neuroinflammation. 2025 Jun 14;22:157. doi: 10.1186/s12974-025-03478-4 (PMC12166563; doi:10.1186/s12974-025-03478-4)
Supplement: Supplementary file 4 — Supplementary Material 4 [file 12974_2025_3478_MOESM4_ESM.docx]

**Supplementary Table S4.** Significantly differed metabolites between control and encephalitis.

| **Name** | **Fold Change (FC)** | **p-value** |
| --- | --- | --- |
| L-Glutamic acid | 0.095527 | 5.15E-09 |
| Pyruvic acid | 0.24398 | 8.36E-09 |
| Butyrylcarnitine | 0.32279 | 5.82E-08 |
| Oxoglutaric acid | 0.28048 | 1.16E-07 |
| L-Proline | 0.285 | 2.20E-07 |
| Thymidine | 0.18239 | 3.01E-07 |
| Malic acid | 0.18839 | 3.71E-07 |
| N-Acetyl-glucosamine 1-phosphate | 0.12446 | 5.77E-07 |
| L-Kynurenine | 0.22008 | 7.26E-07 |
| Carnosine | 0.059394 | 7.49E-07 |
| L-Acetylcarnitine | 0.21484 | 1.05E-06 |
| 4-Trimethylammoniobutanoic acid | 0.26268 | 1.25E-06 |
| Glycerophosphocholine | 0.17908 | 1.56E-06 |
| Niacinamide | 0.16382 | 1.82E-06 |
| Fructose 1,6-bisphosphate | 0.050317 | 1.98E-06 |
| Cytidine monophosphate | 0.084288 | 2.42E-06 |
| Deoxycytidine | 0.10501 | 2.61E-06 |
| Glycine | 0.34085 | 5.15E-06 |
| L-Aspartic acid | 0.10602 | 5.19E-06 |
| Propionylcarnitine | 0.31529 | 6.72E-06 |
| Adenosine monophosphate | 0.06858 | 8.11E-06 |
| 3-Phosphoglyceric acid | 0.03303 | 8.11E-06 |
| Adenosine | 0.095132 | 9.79E-06 |
| Citicoline | 0.111 | 1.01E-05 |
| Allantoin | 0.27351 | 1.06E-05 |
| L-Carnitine | 0.44073 | 1.09E-05 |
| Purine | 0.14699 | 1.16E-05 |
| Phosphoenolpyruvic acid | 0.087968 | 1.27E-05 |
| Guanosine | 0.14075 | 1.36E-05 |
| D-Glyceraldehyde 3-phosphate | 0.057199 | 1.49E-05 |
| Orotic acid | 0.3972 | 1.55E-05 |
| Hexanoylcarnitine | 0.25237 | 1.68E-05 |
| D-Erythrose 4-phosphate | 0.040042 | 1.84E-05 |
| Uridine 5'-monophosphate | 0.10767 | 2.14E-05 |
| Uracil | 0.23348 | 2.46E-05 |
| Fumaric acid | 0.28141 | 2.56E-05 |
| Inosine | 0.099969 | 2.60E-05 |
| Cytosine | 0.20354 | 2.67E-05 |
| Choline | 0.44128 | 3.22E-05 |
| Taurine | 0.15443 | 3.65E-05 |
| D-Sedoheptulose 7-phosphate | 0.054493 | 3.83E-05 |
| S-Adenosylmethionine | 0.15002 | 4.24E-05 |
| Glucose 6-phosphate | 0.053454 | 5.48E-05 |
| Kynurenic acid | 0.16058 | 5.54E-05 |
| ADP | 0.079996 | 5.91E-05 |
| D-Ribulose 5-phosphate | 0.074141 | 6.87E-05 |
| Isovalerylcarnitine | 0.38017 | 7.77E-05 |
| Phosphorylcholine | 0.13842 | 7.88E-05 |
| L-Cystine | 0.46688 | 7.94E-05 |
| Fructose 6-phosphate | 0.073773 | 0.00011 |
| Oxidized glutathione | 0.070523 | 0.000112 |
| CDP-Ethanolamine | 0.088009 | 0.000114 |
| 1-Methylnicotinamide | 0.22508 | 0.000117 |
| D-Ribose 5-phosphate | 0.072124 | 0.000121 |
| Uridine diphosphate-N-acetylglucosamine | 0.1135 | 0.000146 |
| Guanidoacetic acid | 0.28736 | 0.000203 |
| Thiamine monophosphate | 0.16667 | 0.000213 |
| Xanthine | 0.46411 | 0.000337 |
| Inosinic acid | 0.082805 | 0.000355 |
| Guanosine monophosphate | 0.066803 | 0.000481 |
| Hypotaurine | 0.15467 | 0.0005 |
| Citrulline | 0.46293 | 0.000793 |
| Hypoxanthine | 0.32099 | 0.000902 |
| Caprylic acid | 0.45016 | 0.001191 |
| Argininosuccinic acid | 0.42303 | 0.001276 |
| O-Phosphoethanolamine | 0.33995 | 0.00137 |
| Xanthosine | 0.46555 | 0.001776 |
| Pantothenic acid | 0.23329 | 0.001822 |
| Uridine diphosphate glucose | 0.063401 | 0.002002 |
| Thiamine | 0.26382 | 0.004115 |
| S-Adenosylhomocysteine | 0.38529 | 0.005383 |
| Glutathione | 0.086049 | 0.009282 |
| Glycerol 3-phosphate | 0.29116 | 0.01112 |
| N-Acetyl-L-aspartic acid | 0.37226 | 0.025597 |
| 3-Methylhistidine | 2.1265 | 0.030979 |
| Indoxyl sulfate | 0.20751 | 0.042009 |
| D-Ribulose | 0.42221 | 0.043962 |
