# Supplementary material for: Changes in metabolite profiles in the cerebrospinal fluid and in human neuronal cells upon tick-borne encephalitis virus infection
Source: J Neuroinflammation. 2025 Jun 14;22:157. doi: 10.1186/s12974-025-03478-4 (PMC12166563; doi:10.1186/s12974-025-03478-4)
Supplement: Supplementary file 5 — Supplementary Material 5 [file 12974_2025_3478_MOESM5_ESM.docx]

**Supplementary Table S5.** Significantly differed between control and meningitis.

| **Name** | **Fold Change (FC)** | **p-value** |
| --- | --- | --- |
| Pyruvic acid | 0.21194 | 1.52E-10 |
| Butyrylcarnitine | 0.29789 | 5.16E-09 |
| Oxoglutaric acid | 0.26539 | 3.21E-08 |
| L-Proline | 0.28481 | 2.61E-06 |
| Isovalerylcarnitine | 0.39369 | 5.93E-06 |
| Propionylcarnitine | 0.29016 | 7.36E-06 |
| Orotic acid | 0.43297 | 9.38E-06 |
| L-Acetylcarnitine | 0.26569 | 1.75E-05 |
| Glycine | 0.37105 | 1.84E-05 |
| L-Glutamic acid | 0.15539 | 4.56E-05 |
| L-Kynurenine | 0.077474 | 4.71E-05 |
| L-Cystine | 0.48839 | 8.58E-05 |
| Hexanoylcarnitine | 0.26758 | 0.000149 |
| Kynurenic acid | 0.17817 | 0.000241 |
| N-Acetyl-glucosamine 1-phosphate | 0.20227 | 0.000411 |
| Fructose 1,6-bisphosphate | 0.1605 | 0.000509 |
| S-Adenosylmethionine | 0.3791 | 0.00051 |
| D-Erythrose 4-phosphate | 0.097366 | 0.001175 |
| Malic acid | 0.2605 | 0.001806 |
| L-Aspartic acid | 0.19655 | 0.002209 |
| ADP | 0.16045 | 0.002245 |
| 4-Trimethylammoniobutanoic acid | 0.34412 | 0.002608 |
| Thymidine | 0.24171 | 0.00311 |
| Inosinic acid | 0.12704 | 0.003675 |
| Guanosine monophosphate | 0.064715 | 0.004075 |
| Cytidine monophosphate | 0.1256 | 0.00445 |
| Glucose 6-phosphate | 0.12843 | 0.004722 |
| Adenosine monophosphate | 0.070535 | 0.005172 |
| D-Glyceraldehyde 3-phosphate | 0.11732 | 0.005215 |
| 3-Phosphoglyceric acid | 0.099683 | 0.005304 |
| Taurine | 0.25631 | 0.006013 |
| D-Ribulose 5-phosphate | 0.16066 | 0.006917 |
| Adenosine | 0.078393 | 0.006976 |
| Carnosine | 0.1083 | 0.007642 |
| Citicoline | 0.11722 | 0.00776 |
| D-Sedoheptulose 7-phosphate | 0.10934 | 0.007999 |
| Uridine diphosphate glucose | 0.069097 | 0.008379 |
| Oxidized glutathione | 0.13899 | 0.008606 |
| Cytosine | 0.27947 | 0.008733 |
| CDP-Ethanolamine | 0.13446 | 0.00929 |
| Guanosine | 0.22471 | 0.011875 |
| Fructose 6-phosphate | 0.14968 | 0.012632 |
| Deoxycytidine | 0.15493 | 0.013011 |
| Fumaric acid | 0.34411 | 0.01447 |
| O-Phosphoethanolamine | 0.45124 | 0.015682 |
| Uridine 5'-monophosphate | 0.10596 | 0.015881 |
| Allantoin | 0.35676 | 0.017334 |
| Glycerophosphocholine | 0.15682 | 0.017402 |
| Niacinamide | 0.22344 | 0.018716 |
| Glutathione | 0.33632 | 0.020775 |
| Uracil | 0.32853 | 0.022419 |
| Xanthosine | 0.49515 | 0.024096 |
| Phosphoenolpyruvic acid | 0.21752 | 0.024994 |
| Choline | 0.46708 | 0.025212 |
| Uridine diphosphate-N-acetylglucosamine | 0.1493 | 0.025478 |
| Inosine | 0.18041 | 0.028154 |
| Indoxyl sulfate | 0.34023 | 0.029868 |
| D-Ribose 5-phosphate | 0.19186 | 0.033657 |
| S-Adenosylhomocysteine | 0.4373 | 0.036089 |
| Purine | 0.22595 | 0.037986 |
| Hypotaurine | 0.20904 | 0.046113 |
