# Supplementary material for: Changes in metabolite profiles in the cerebrospinal fluid and in human neuronal cells upon tick-borne encephalitis virus infection
Source: J Neuroinflammation. 2025 Jun 14;22:157. doi: 10.1186/s12974-025-03478-4 (PMC12166563; doi:10.1186/s12974-025-03478-4)
Supplement: Supplementary file 6 — Supplementary Material 6 [file 12974_2025_3478_MOESM6_ESM.docx]

**Supplementary Table S6.** Biomarker panel.

| **Name** | **AUC** | **p-value** |
| --- | --- | --- |
| S-Adenosylmethionine | 0.81333 | 0.0016348 |
| 1-Methylnicotinamide | 0.78 | 6.835E-4 |
| Phosphoenolpyruvic acid | 0.75667 | 0.0031707 |
| Fructose 1,6-bisphosphate | 0.74833 | 0.0047236 |
| 3-Phosphoglyceric acid | 0.74167 | 0.0049862 |
| D-Ribose 5-phosphate | 0.73 | 0.0084463 |
| Cytidine | 0.71833 | 0.0082922 |
| L-Arginine | 0.71667 | 0.014023 |
| Caprylic acid | 0.715 | 0.015646 |
| Thymidine | 0.715 | 0.025621 |
| Hypoxanthine | 0.71333 | 0.010689 |
