# Supplementary material for: Changes in metabolite profiles in the cerebrospinal fluid and in human neuronal cells upon tick-borne encephalitis virus infection
Source: J Neuroinflammation. 2025 Jun 14;22:157. doi: 10.1186/s12974-025-03478-4 (PMC12166563; doi:10.1186/s12974-025-03478-4)
Supplement: Supplementary file 7 — Supplementary Material 7 [file 12974_2025_3478_MOESM7_ESM.docx]

**Supplementary Fig S1. Human anti-inflammatory cytokine IL-10 profiling in the cerebrospinal fluid of hospitalised TBEV patients and controls.** IL-10 level was assayed by ELISA. Significance was determined using a one-way ANOVA followed by Bonferroni multiple comparison test (**p* < 0.05, ***p* < 0.01 ****p* < 0.001, *****p* < 0.0001); ns, not significant. Data are the mean ± standard deviation of 3 technical repeats and 2 independent experiments.
