# Supplementary material for: Changes in metabolite profiles in the cerebrospinal fluid and in human neuronal cells upon tick-borne encephalitis virus infection
Source: J Neuroinflammation. 2025 Jun 14;22:157. doi: 10.1186/s12974-025-03478-4 (PMC12166563; doi:10.1186/s12974-025-03478-4)
Supplement: Supplementary file 8 — Supplementary Material 8 [file 12974_2025_3478_MOESM8_ESM.docx]

**Supplementary Fig S2. Sinefungin, MB05032 and NaF reduce TBEV release. (A)** ELISA analysis of soluble S-Adenosylmethionine (SAM), and Fructose 1,6-bisphosphate (FBP1) and Phosphoenolpyruvic acid (PEP) levels from hMNs treated with Sinefungin, MB05032 [0.0, 0.1, and 1.0 µM], NaF [0.0, 0.1, and 1.0 mM]) for 24 h. hMNs were treated with increasing concentrations of **(B)** Sinefungin, **(C)** MB05032, **(D)** NaF for 24 h and infected with TBEV at an MOI of 1 PFU, plaque-forming units. Viral titres at 24 and 48 hpi were determined via plaque assay. **(E)** Quantification of extracellular glucose (left) and intracellular glucose-6-phosphate (G6P, right) in uninfected vs. TBEV-infected cells at 24 hpi. **(F)** As in (E), but with MB05032 (1 µM) treatment. Statistical significance was determined using a one-way ANOVA followed by Bonferroni multiple comparison test (**p* < 0.05, ***p* < 0.01 ****p* < 0.001, *****p* < 0.0001); ns, not significant. Data are the mean ± standard deviation of 4 technical repeats in 3 independent experiments.
