# Supplementary figures and images for: Changes in metabolite profiles in the cerebrospinal fluid and in human neuronal cells upon tick-borne encephalitis virus infection
Source: J Neuroinflammation. 2025 Jun 14;22:157. doi: 10.1186/s12974-025-03478-4 (PMC12166563; doi:10.1186/s12974-025-03478-4)

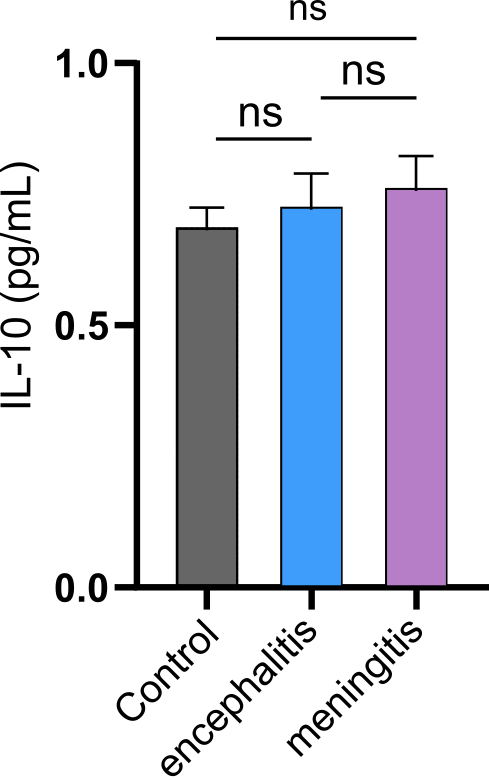

Supplement: Supplementary file 9 — Supplementary Material 9 [file 12974_2025_3478_MOESM9_ESM.png]

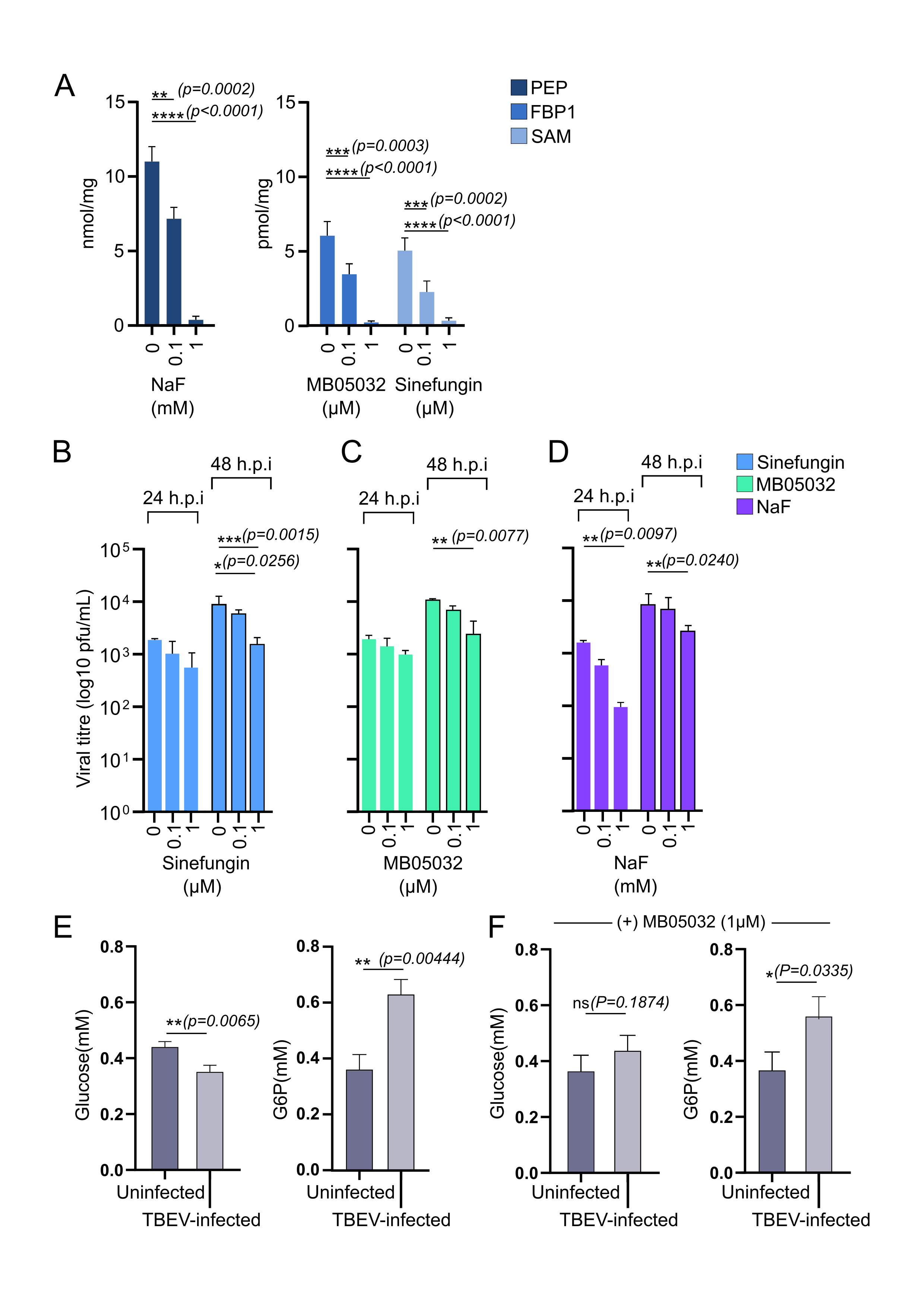

Supplement: Supplementary file 10 — Supplementary Material 10 [file 12974_2025_3478_MOESM10_ESM.png]
